# Embryonic lethality and defective mammary gland development of activator‐function impaired conditional knock‐in *Erbb3* ^*V943R*^ mice

**DOI:** 10.1002/ggn2.10036

**Published:** 2020-12-05

**Authors:** Kate Senger, Wenlin Yuan, Meredith Sagolla, Jonas Doerr, Brad Bolon, James Ziai, Kai‐Hui Sun, Soren Warming, Merone Roose‐Girma, Na Zhang, Lucinda Tam, Robert J. Newman, Subhra Chaudhuri, Aju Antony, Leonard D. Goldstein, Steffen Durinck, Bijay S. Jaiswal, Daniel Lafkas, Zora Modrusan, Somasekar Seshagiri

**Affiliations:** ^1^ Department of Molecular Biology Genentech South San Francisco California USA; ^2^ Department of Pathology Genentech South San Francisco California USA; ^3^ GEMpath Inc. Longmont Colorado USA; ^4^ SciGenom Labs Pvt Ltd Kochi Kerala India; ^5^ Department of Bioinformatics and Computational Biology Genentech South San Francisco California USA; ^6^ Department of Immunology Discovery Genentech South San Francisco California USA; ^7^ SciGenom Research Foundation Bangalore Karnataka India

**Keywords:** animal genetics, basic medical sciences, developmental genetics, genetics, genetics or genomics, medicine, molecular medicine

## Abstract

ERBB3 is a pseudokinase domain‐containing member of the ERBB family of receptor tyrosine kinases (RTKs). Following ligand binding, ERBB receptors homo‐ or hetero‐dimerize, leading to a head‐to‐tail arrangement of the intracellular kinase domains, where the “receiver” kinase domain of one ERBB is activated by the “activator” domain of the other ERBB in the dimer. In ERBB3, a conserved valine at codon 943 (V943) in the kinase C‐terminal domain has been shown to be important for its function as an “activator” kinase *in vitro*. Here we report a knock‐in mouse model where we have modified the endogenous *Erbb3* allele to allow for tissue‐specific conditional expression of *Erbb3* ^*V943R*^ (*Erbb3* ^*CKI‐V943R*^). Additionally, we generated an *Erbb3* ^*D850N*^ (*Erbb3* ^*CKI‐D850N*^) conditional knock‐in mouse model where the conserved aspartate in the DFG motif of the pseudokinase domain was mutated to abolish any potential residual kinase activity. While *Erbb3* ^*D850N/D850N*^ animals developed normally, homozygous *Erbb3* ^*V943R/V943R*^ expression during development resulted in embryonic lethality. Further, tissue specific expression of *Erbb3* ^*V943R/V943R*^ in the mammary gland epithelium following its activation using *MMTV‐Cre* resulted in delayed elongation of the ductal network during puberty. Single‐cell RNA‐seq analysis of *Erbb3* ^*V943R/V943R*^ mammary glands showed a reduction in a specific subset of fibrinogen‐producing luminal epithelial cells.

## Graphical abstract

ERBB3 receptor tyrosine kinase, a member of the ERBB family, has an important role in development and disease. It is the only member of the ERBB family with a pseudokinase domain and it preferentially heterodimerizes with ERBB2 to activate ERBB2 through allosteric kinase domain interactions. While targeted replacement of a conserved amino acid that abolishes its residual protein kinase activity was consistent with life and normal development, replacement of a residue essential for activator‐receptor interactions showed embryonic lethality and delayed ductal elongation in the developing mutant mammary gland. Single cell analysis of the targeted mammary glands showed reduction in a specific subset of fibrinogen‐producing luminal epithelial cells, suggesting that these cells are dependent on ERBB3‐mediated allosteric signaling.

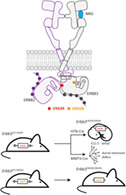

## 1 INTRODUCTION

The human epidermal growth factor receptor (ERBB/HER) family of receptors consists of four members: EGFR/ERBB1/HER1, ERBB2/HER2/NEU1, ERBB3/HER3, and ERBB4/HER4.^1^, ^2^, ^3^ They play an important role in development and disease.^4^ Mutations and amplification and overexpression of ERBB family members drive multiple cancers.^4^, ^5^

The ERBB receptors are comprised of an N‐terminal extracellular ligand‐binding domain, a single‐pass transmembrane domain, an intracellular receptor tyrosine kinase (RTK) domain, and a tyrosine rich C‐terminal signaling tail. In the inactive state, the extracellular domain (ECD) of ERBB receptors exists in a compact folded “closed” conformation. Ligands that bind to ERBB family members include the epidermal growth factor (EGF), transforming growth factor‐α (TGF‐α), and neuregulins 1‐4 (NRG1‐NRG4).^6^ Following ligand binding, the ERRB receptors' ECD adopts an “open” conformation enabling hetero‐ or homodimerziation and signaling.^7^ ERBB2, with no known ligand, normally adopts an “open” conformation and preferentially heterodimerizes with other ligand bound family members to signal.

EGFR, the prototype ERBB family member, upon ligand binding adopts an open conformation leading to homodimerization of its ECD and a head‐to‐tail asymmetric arrangement of the intracellular kinase domains, leading to their activation.^8^ Structural studies identified a valine (V) at codon 924 in the C‐terminal lobe of the “activator” kinase that is critical for its interaction with the N‐terminal lobe of the “receiver” kinase, leading to its allosteric activation.^8^, ^9^ Mutating valine 924 to an arginine (V924R) abolished the “activator” function of EGFR kinase.^8^

Though the ERBB3 intracellular pseudokinase domain can bind ATP, it lacks residues critical for its phosphorylation activity.^10^ Consistent with this, ERBB3 has been shown to be kinase‐dead or have a very weak kinase activity.^11^, ^12^ ERBB3 following ligand engagement preferentially heterodimerizes with ERBB2, leading to ERBB2 activation.^13^, ^14^ In the ERBB3‐ERBB2 heterodimer, the ERBB3 pseudokinase domain functions solely as an allosteric “activator” while the ERBB2 kinase domain functions as the “receiver”.^15^ Mutating the conserved valine at codon 943 (equivalent of EGFR V924) to arginine (V943R), within the C‐terminal activator interface of ERBB3, impaired its ability to activate EGFR kinase *in vitro*.^16^, ^17^

ERBB3, though it lacks a competent kinase‐active domain, plays an important role in development and disease.^4^ Mouse knock‐out studies show that a majority of the *Erbb3* ^*−/−*^ null mice die between E11.5 and E13.5 and show extensive cardiac developmental defects.^18^, ^19^, ^20^, ^21^ Additionally, *Erbb3* ^*−/−*^ null embryos showed neural defects and poor differentiation of organs that include stomach, pancreas and adrenal glands.^18^, ^19^ Mammary gland‐specific targeted *ErbB3* knock‐out mice, PI3K‐signaling impaired *ErbB3* mutant knock‐in mice (*Erbb3* ^*Δ85/Δ85*^), and an *ErbB3* ^−/−^ embryo derived mammary transplant model reported defects in ductal morphogenesis and lactation.^20^, ^22^, ^23^, ^24^, ^25^

*ERBB3* amplification, overexpression, and mutations contribute to the development, progression and acquired drug resistance in human cancers.^4^, ^26^ *ERBB3* knock‐down in *ERBB2‐*amplified mammary cancer cells blocked tumor formation.^27^ Further, tissue‐specific deletion of *Erbb3* impaired colon,^21^ liver,^28^ and mammary tumor development.^29^

Targeting ERBB3 using antibodies or small molecule inhibitors for treating cancer remains elusive.^26^ To confirm the relevance of ERBB3 activator function *in vivo,* and also generate a resource that can be used to further clarify the role of ERBB3 in cancer, we generated activator‐function impaired V943R mutant *Erbb3* mice. We also generated D850N knock‐in mice to test the relevance of any residual ERBB3 kinase activity for development. To generate the activator mutant, we modified the endogenous *Erbb3* allele by placing a dormant copy of *Erbb3* exon 23 encoding V943R. The engineered mice expressed wild‐type *Erbb3* from the modified conditional knock‐in (*Erbb3* ^*CKI‐V943R*^) locus prior to activation of the dormant mutant allele. Upon Cre‐mediated recombination, the mutant *ErbB3* ^*V943R*^ allele was conditionally activated, leading to its expression. Activation of *Erbb3* ^*V943R*^ mutant allele expression during development resulted in embryonic lethality at around E12.0. Histopathological analysis of the embryos revealed defects in the heart that include thinning myocardium and diminished endocardial cushions. Further, tissue‐specific expression of the *Erbb3* ^*V943R*^ mutant allele in developing mammary glands using an *MMTV‐Cre* driver led to a delay in duct elongation and structural changes to the terminal end buds. Consistent with this, single‐cell RNA‐sequencing of luminal epithelial cells from pubescent mice revealed a subset of mature luminal cells that require ERBB3 signaling for survival. Unlike V943R mice, the kinase‐dead D850N mice (*Erbb3* ^*D850N/D850N*^) were viable and developed normally, indicating that the residual kinase activity of ERBB3 is dispensable for normal development. These data indicate that though the residual kinase activity of ERBB3 is dispensable, its activator function is indispensable for normal development and cellular signaling.

## 2 RESULTS

### 2.1 Allosteric activation of ERBB2 is impaired in V943R activator interface mutant ERBB3

**FIGURE 1:**
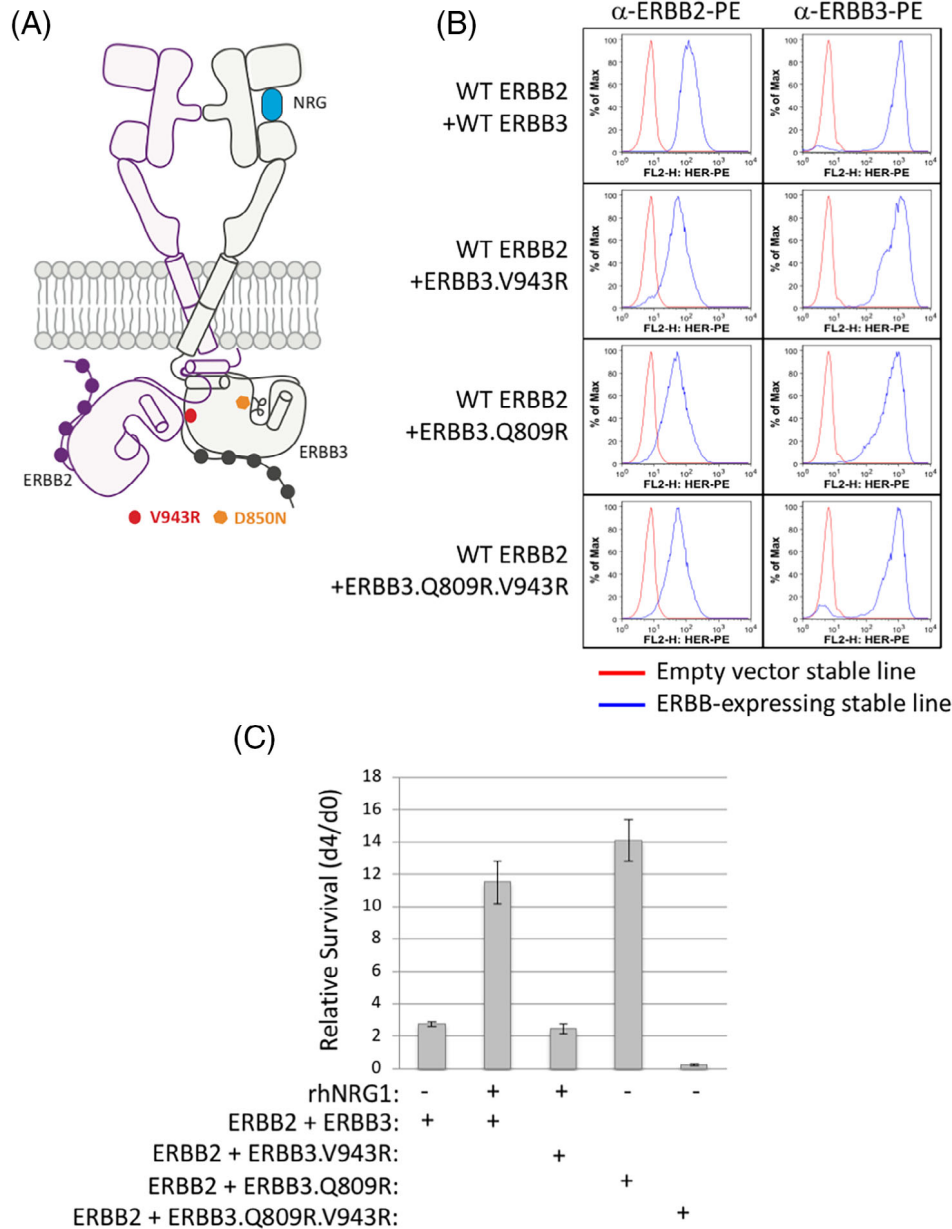
The V943R ERBB3 activator‐interface mutation impairs ERBB2 activation. A, Cartoon depicting ERBB3‐ERBB2 heterodimer. The activator‐interface mutation V943R and kinase dead D850N mutation are denoted on ERBB3. B, Cell surface expression of indicated ERBB2 and ERBB3 WT or variants in Ba/F3 stable cell lines measured using FACS Calibur flow cytometer. Empty vector (red) or the indicated ERBB expression constructs (blue) were incubated with PE‐conjugated antibodies against ERBB2 or ERBB3. C, Relative survival of Ba/F3 stable cell lines expressing the indicated constructs. NRG, neuregulin ligand

We used the IL‐3 dependent Ba/F3 pro‐B cell line to assess the function of the ERBB3 V943R activator‐interface mutant, as described previously^30^ (Figure 1A and Figure S1). Briefly, we generated Ba/F3 cells stably expressing ERBB3 V943R or other indicated mutations in combination with ERBB2 and confirmed the surface expression of the receptors by flow cytometry (Figure 1B). We tested cell survival following interleukin‐3 (IL‐3) withdrawal (Figure 1C). As expected, we found that stimulation of Ba/F3 cells, stably co‐expressing wild‐type (WT) human ERBB3 and ERBB2, with the ERBB3 ligand neuregulin‐1 (NRG1) promoted IL‐3 independent cell survival (Figure 1C). In contrast, co‐expression of ERBB3 V943R and WT ERBB2 in the presence of NRG1 did not support IL‐3 independent cell survival. Previously, co‐expression of an ERBB3 Q809R oncogenic mutant along with WT ERBB2 in Ba/F3 cells, in the absence of ligand NRG1, was shown to promote IL‐3 independent survival.^31^ We found that ErbB3 Q809R V943R, a double mutant that carried the activator interface mutation, when co‐expressed with WT ERBB2, did not support IL‐3 independent survival of Ba/F3 cells, further confirming the requirement of V943 for its “activator” function. Consistent with the cell survival activity observed in the Ba/F3 assay, in the absence of IL‐3, WT ERBB2‐ and WT ERRB3‐expressing cells treated with NRG1 showed elevated p‐ERBB2, p‐ERBB3, p‐Akt and p‐ERK‐1/2, indicating downstream signaling and activation (Figure S2). However, cells expressing ERBB3 V943R mutant in combination with ERBB2 failed to show downstream activation markers (Figure S2). ERBB2 levels were reduced in ERBB3 V943R mutant lines, indicating that ERBB3, apart from contributing to ERBB2 activation and signaling, perhaps affect its stability and/or turnover (Figure S2).

### 2.2 Generation of conditionally activatable *ErbB3* ^*CKI‐V943R*^ knock‐in mice

**FIGURE 2:**
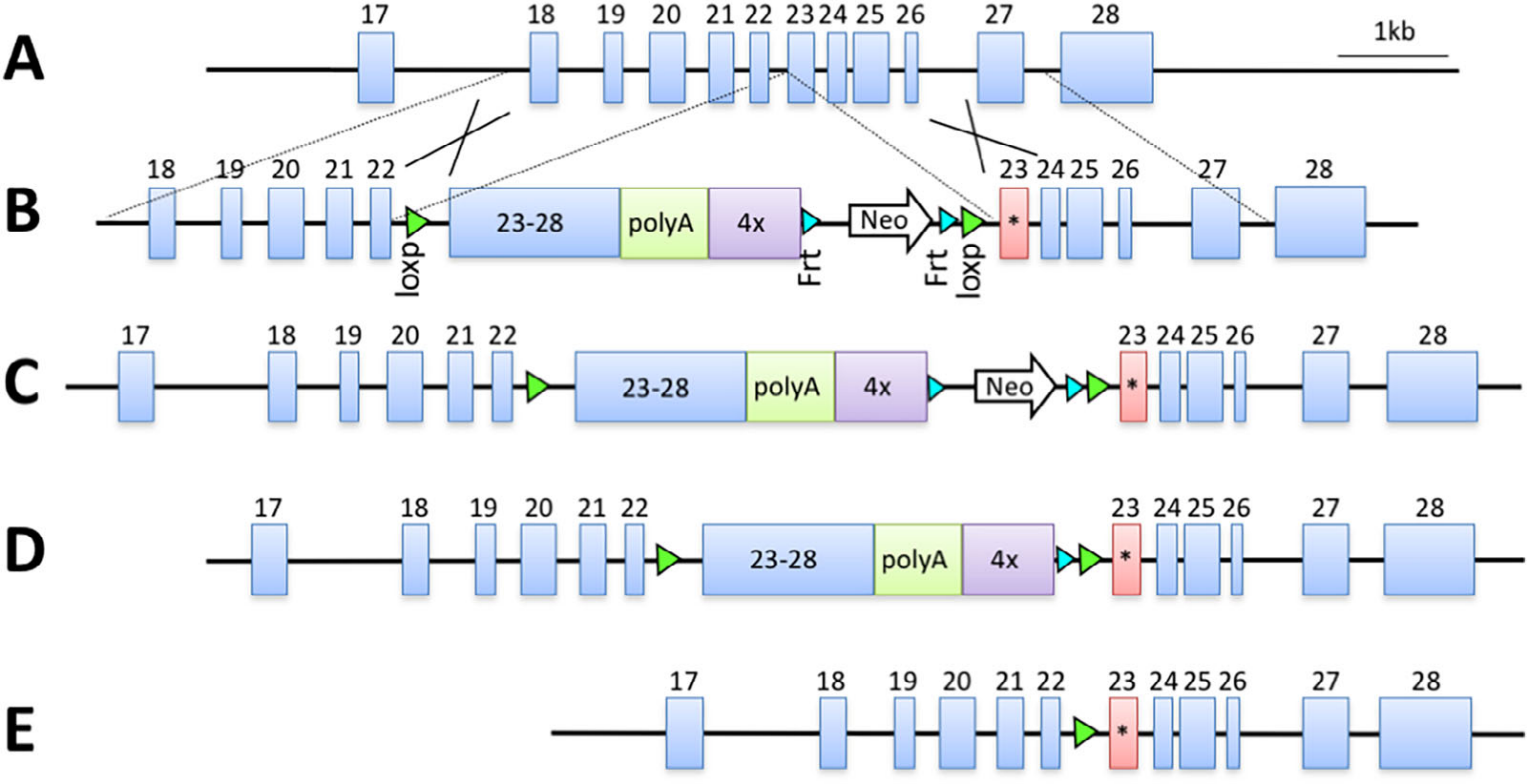
Generation of mice with a conditional knock‐in *Erbb3* ^*CKI‐V943R*^ allele. A, Genomic region of the *Erbb3* gene. B, Targeting vector used to modify the *Erbb3* locus. Homology arms for targeting of C57BL/6N ES cells are indicated. An asterisk marks the location of the V943R mutation. C, Targeted *Erbb3* locus with *Neo* selection marker present. D, *Erbb3* ^*CKI‐V943R*^ conditional knock‐in allele after FLP‐mediated excision of *Neo*. E, *Erbb3* ^*V943R*^ knock‐in allele after Cre‐mediated excision of the floxed wildtype *Erbb3* cassette

To study the activator function of ERBB3 *in vivo*, we engineered a mouse that can conditionally express an activator‐function deficient *Erbb3* ^*V943R*^ allele under its native promoter (Figure 2). In this genetically engineered mouse model we replaced the WT exon 23 (Figure 2A) in embryonic stem cells with a targeting vector composed of a *LoxP* flanked cDNA minicassette coding for exon 23‐28, a polyA signal sequence and a transcriptional stop cassette (Figure 2B). The modified wild‐type exon 23 was followed by a copy of *Erbb3* exon 23 encoding the V943R mutation (Figure 2C). The conditional knock‐in (CKI) allele *Erbb3* ^*CKI‐V943R*^ was created after FLP‐mediated excision of the neomycin (Neo) cassette (Figure 2D). Expression of the mutant allele was induced by Cre recombinase (Figure 2E).

### 2.3 Activator function‐impaired *Erbb3* ^*V943R*^ ^*/V943R*^ expression results in embryonic lethality

**FIGURE 3:**
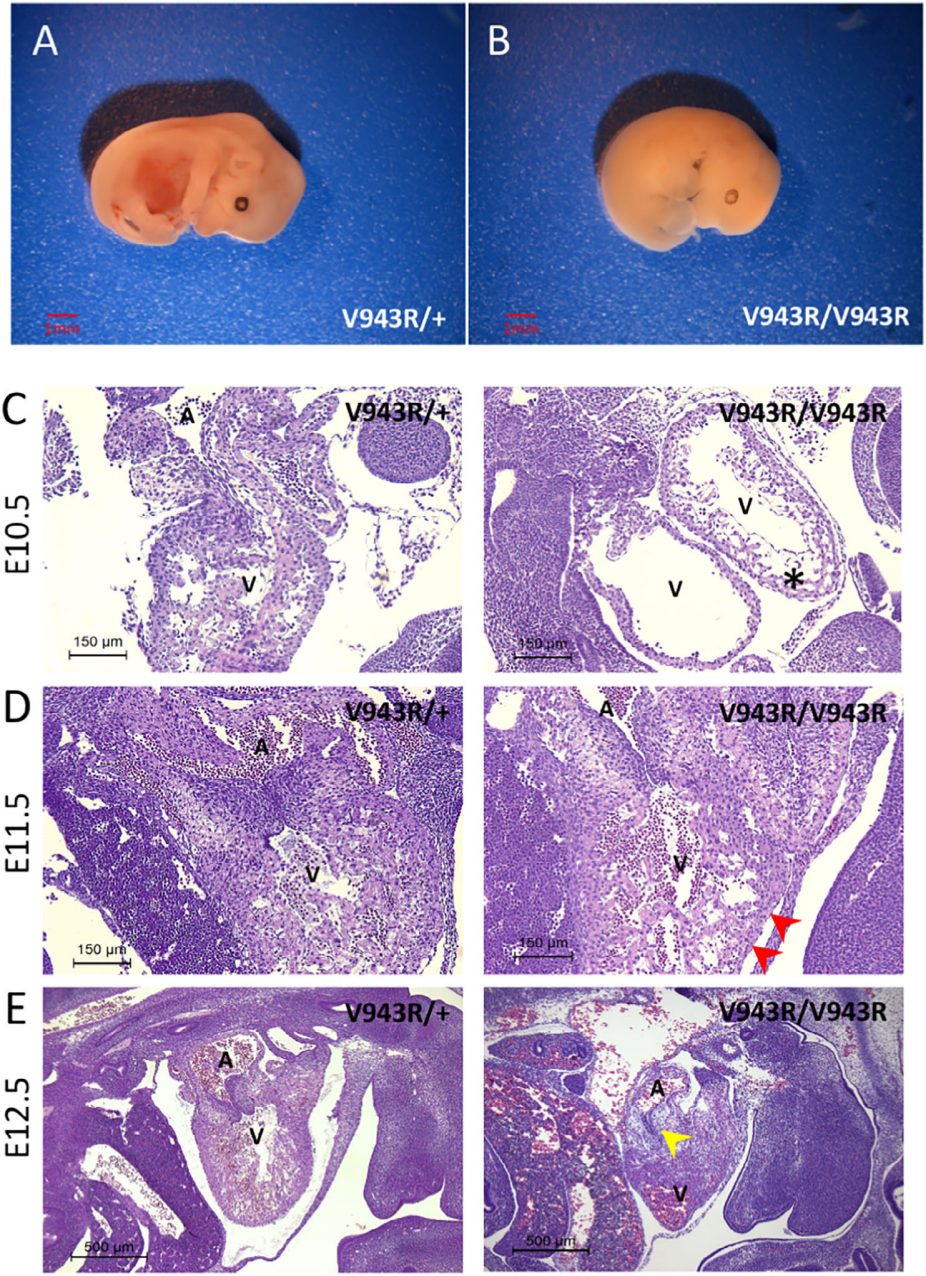
*Erbb3* ^*V943R/V943R*^ embryos die prenatally and display defects during early cardiac development. A,B, *Erbb3* ^*V943R/V943R*^ mice were generated by treating *Erbb3* ^*CKI‐V943R/+*^ zygotes with recombinant HTN‐Cre followed by intercrossing. Control *Erbb3* ^*V943R/+*^ embryos, A, and age‐matched *Erbb3* ^*V943R/V943R*^ embryos, B, were harvested at E10.5, E11.5, E12.5, and E13.5. The estimated time of death of the *Erbb3* ^*V943R/V943R*^ embryos was approximately E12.0, based on the absence of digital rays on the forelimbs and white surface features. C‐E, One representative *Erbb3* ^*V943R/+*^ embryo (left panels) and *Erbb3* ^*V943R/V943R*^ embryo (right panels) from each time point was fixed, sectioned, and stained with hematoxylin and eosin (H&E) for histopathological assessment. At E10.5, C, the *Erbb3* ^*V943R/V943R*^ ventricular walls and trabeculae are smaller than expected (star), have fewer nuclei, and show cardiomyocyte anisocytosis, and increased clear connective tissue beneath the endocardium. At E11.5, D, *Erbb3* ^*V943R/V943R*^ ventricular walls appear thin and the clearness of the subendocardial tissue is minimal (arrowheads). At E12.5, E, diminished endocardial cushions are clearly apparent in *Erbb3* ^*V943R/V943R*^ embryos (arrowhead). A, atrium; V, ventricle

To induce whole animal expression of the mutant V943R allele we treated developing *Erbb3* ^*CKI‐V943R/+*^ embryos with the cell‐permeant HTN‐Cre recombinase at E0.5. In intercrosses between *Erbb3* ^*CKI‐V943R/+*^; HTN‐Cre mice we did not observe any homozygous *Erbb3* ^*V943R/V943R*^ animals, although we identified *Erbb3* ^*+/+*^ and *Erbb3* ^*V943R/+*^ offspring (Table S1 and Figure S3A). The observed deviation from the expected Mendelian ratio for the *Erbb3* ^*V943R/V943R*^ homozygous animals suggested embryonic lethality. To confirm this, we performed timed pregnancy studies in which the mating period was constrained to a 2 hours window rather than overnight. We harvested embryos at E10.5, E11.5, E12.5, and E13.5 and genotyped them. We then visually compared the external features of the embryos based on their confirmed genotypes (Figure 3A,B). At E13.5, heterozygous CKI (*Erbb3* ^*V943R/+*^) embryos appeared normal with visible red‐brown liver spots in the cranial abdomen, prominent digits on both forelimbs and hind limbs, and thin and tortuous subcutaneous blood vessels. In contrast, the age‐matched homozygous *Erbb3* ^*V943R/V943R*^ embryos were dead and were uniformly white in color except for the presence of the retinal melanin ring (Figure 3B). The estimated time of *in utero* death was approximately E12.0, based on the absence of digital rays on the forelimbs, which typically form at about E12.3.

To determine whether homozygous *Erbb3* ^*V943R/V943R*^ embryos exhibit developmental defects, histopathological assessment was performed on E10.5, E11.5, and E12.5 embryos (Figure 3C‐E). The *Erbb3* ^*V943R/V943R*^ mice had noticeable heart defects, including thinning of the structures formed by ventricular myocardiocytes, expansion of the subendocardial connective tissue, and reduced endocardial cushions (Figures 3C‐E). These defects bear a marked similarity to those seen in *Erbb3* ^*−/−*^ null mice.^18^

### 2.4 ERBB3 activator function is required for developing mammary ductal epithelium elongation

**FIGURE 4:**
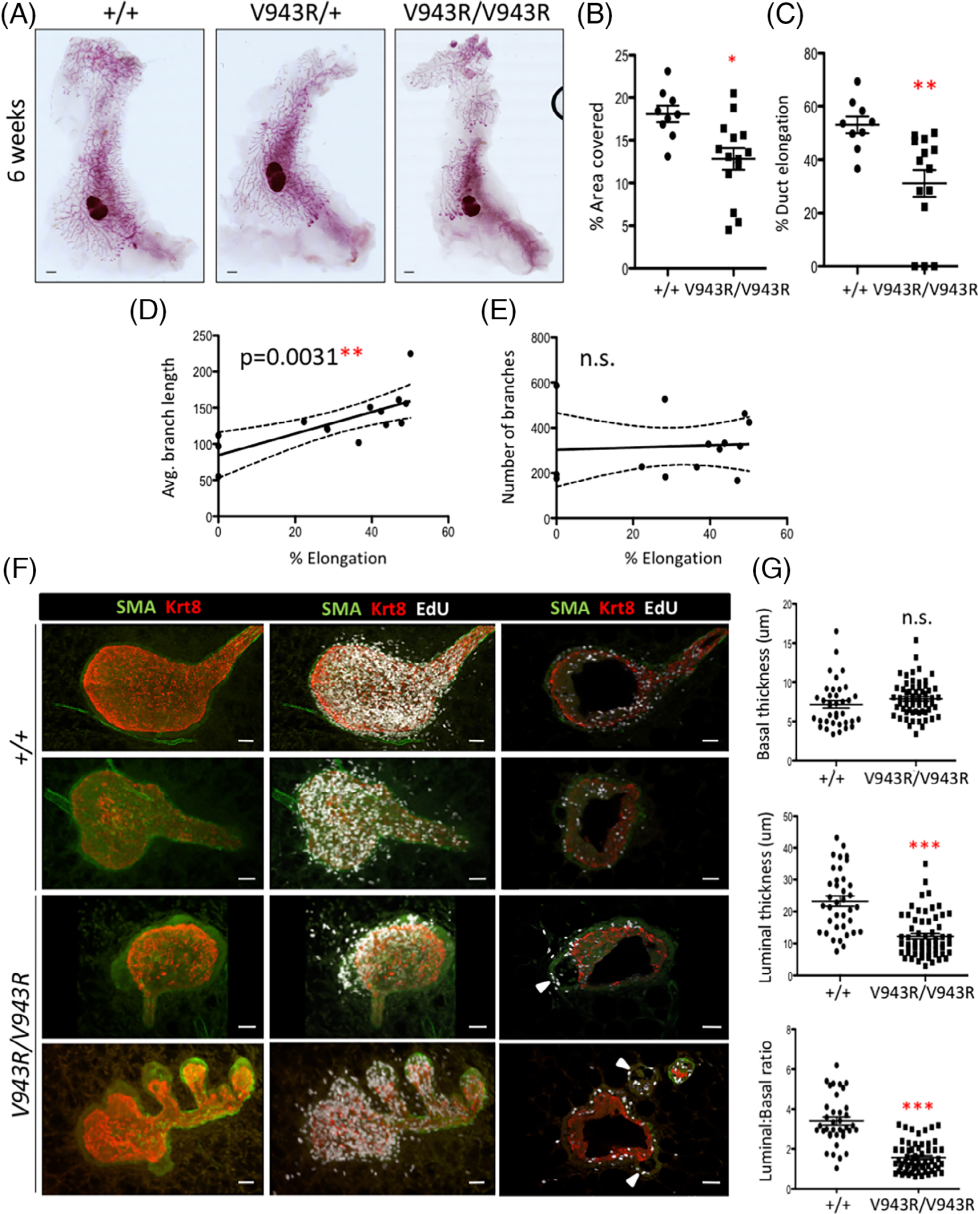
*Erbb3* ^*V943R/V943R;MMTV‐Cre*^ mice show defects in duct elongation and terminal end bud structure. A, Mammary epithelial‐specific knock‐in was achieved by intercrossing mice carrying *Erbb3* ^*CKI‐V943R*^ and *MMTV‐Cre* alleles. At 6 weeks of age, the fourth (abdominal) mammary gland was dissected from mice of the indicated genotypes, fixed in Carnoy's solution, and stained with carmine. Scale bar = 1 mm. B, The percentage of gland area covered by ducts in the region around the lymph node was measured for *Erbb3* ^*+/+;MMTV‐Cre*^ (N = 9) and *Erbb3* ^*V943R/V943R;MMTV‐Cre*^ (N = 14) mice at 6 weeks of age ( *P* = .007). C, Elongation of the ducts as a percentage of total distance from lymph node to end of gland was measured for *Erbb3* ^*+/+;MMTV‐Cre*^ (N = 9) and *Erbb3* ^*V943R/V943R;MMTV‐Cre*^ (N = 14) mice at 6 weeks of age ( *P* = .004). D, The average branch length in 6 week old *Erbb3* ^*V943R/V943R;MMTV‐Cre*^ glands was plotted as a function of percent elongation. E, The number of branches in *Erbb3* ^*V943R/V943R;MMTV‐Cre*^ cohort was plotted as a function of percent elongation. F, Two representative TEBs from 6‐week old female *Erbb3* ^*+/+;MMTV‐Cre*^ and *Erbb3* ^*V943R/V943R;MMTV‐Cre*^ female mice. EdU^+^ cells were stained with AlexaFluor‐647. Smooth muscle actin (SMA, AlexaFluor‐488 secondary) and keratin‐8 (KRT8, AlexaFluor‐555 secondary) were detected using appropriate antibodies. Scale bar = 30 um. G, Measurements of basal (SMA^+^) and luminal (KRT8^+^) thicknesses were taken using Imaris (Bitplane) at 12 or more locations around the circumference of *Erbb3* ^*+/+;MMTV‐Cre*^ (N = 3) and *Erbb3* ^*V943R/V943R;MMTV‐Cre*^ I (N = 5) TEBs. n.s. = not significant; *P* = 2.5 × 10^−9^ for luminal thickness measurements; *P* = 4.0 × 10^−15^ for luminal:basal ratios

ERBB3 has an important role in mammary epithelium development during puberty,^22^, ^23^ We tested the role of ERBB3 activator function in mammary gland development by activating the expression of the *Erbb3* ^*V943R*^ allele in the mammary gland using the mammary epithelial‐specific MMTV‐Cre driver (*Erbb3* ^*V943R/V943R;MMTV‐Cre*^). We first confirmed expression of the V943R mutant allele in mammary epithelial cells using RT‐PCR and sequencing (Figure S3B). Unlike the homozygous *Erbb3* ^*V943R/V943R*^ mice, the *Erbb3* ^*V943R/V943R;MMTV‐Cre*^ homozygous mice were viable and were born at the expected 1:2:1 Mendelian ratio (Table S2). To assess the effect of the ERBB3 V943R mutation, we harvested the abdominal mammary glands during puberty (6 weeks) and stained them with carmine (Figure 4A). At 6 weeks of age, *Erbb3* ^*+/+;MMTV‐Cre*^ (n = 9) and *Erbb3* ^*V943R/+;MMTV‐Cre*^ (n = 5) mammary glands had ducts extending approximately halfway from the inguinal lymph node to the end of the gland. However, homozygous *Erbb3* ^*V943R/V943R;MMTV‐Cre*^ mammary glands (n = 14) had reduced duct extension.

To quantify the extent of the mammary ductular defect in *Erbb3* ^*V943R/V943R;MMTV‐Cre*^ mice, we performed high‐resolution scans of the glands and delineated the ductal network using the ImageJ software (Figure S4A‐C). To quantify the extent to which the ducts covered the gland, we designated a region of interest (ROI) centered on the inguinal lymph node and measured the percent area covered by ducts by 6 weeks of age (Figure 4B). On average, *Erbb3* ^*+/+;MMTV‐Cre*^ mice showed 18% coverage, while *Erbb3* ^*V943R/V943R;MMTV‐Cre*^ mice showed 12% coverage, with 3 out of the 14 *Erbb3* ^*V943R/V943R;MMTV‐Cre*^ glands showing as little as ~5% coverage. We also measured the distance of duct elongation as a percentage of a reference line drawn between the center of the inguinal lymph node to the end of the gland (Figure 4C). By 6 weeks of age the ducts from *Erbb3* ^*+/+;MMTV‐Cre*^ mice had elongated an average of 53% of this distance, while ducts from *Erbb3* ^*V943R/V943R;MMTV‐Cre*^ had only migrated an average of 22% of the distance. For three of these glands there was no elongation of the ducts past the lymph node. Interestingly, we observed differences in elongation distance within abdominal mammary glands dissected from the same *Erbb3* ^*V943R/V943R;MMTV‐Cre*^ mouse (Figure S4D), demonstrating variability of the phenotype on a per gland basis. By 12 weeks of age, a timepoint at which the mammary fat pad is typically infiltrated completely with ducts, we observed that most of the *Erbb3* ^*V943R/V943R;MMTV‐Cre*^ mice (11 of 13) did not appear to have a defect, while 2 out of 13 mice (15%) displayed shortened ducts (Figure S4E). The observation that most animals display fully elongated ducts by 12 weeks of age suggests that the defect seen at 6 weeks represents a delay in elongation rather than a permanent defect.

In addition to measuring elongation, we also quantified the number of branches in the *Erbb3* ^*V943R/V943R;MMTV‐Cre*^ glands to determine whether or not the ERBB3 V943R substitution affected branching morphogenetic programs (Figure 4D,E). As expected, average branch length was significantly correlated with percent elongation of the entire ductal network (*P* = .0031, Figure 4D). We did not, however, detect a change in branch number as a function of elongation in the *Erbb3* ^*V943R/V943R;MMTV‐Cre*^ glands (Figure 4E). Taken together these results suggest ERBB3 V943R delays elongation but does not impact branching developmental programs within the developing ductal network.

To evaluate whether or not the overall percentages of basal and luminal epithelial cell populations were altered in the *Erbb3* ^*V943R/V943R;MMTV‐Cre*^ ductal network, we used flow cytometry on enzyme‐digested mammary glands (Figure S5A‐D). We did not detect any significant changes in the percentage of either luminal or basal cells across genotypes, nor in the overall ratio of luminal to basal epithelial cells.

We next examined the local cellular organization of the TEBs in more detail. Terminal end buds (TEBs) are bulbous structures at the ends of ducts that appear during puberty and drive morphological changes in the ductal network during this time. TEBs are comprised of a single layer of basal myoepithelial cells encasing a mass of proliferating luminal epithelial cells. *Erbb3* ^*+/+;MMTV‐Cre*^ and *Erbb3* ^*V943R/V943R;MMTV‐Cre*^ (n = 3 per genotype) mice were injected intraperitoneally with ethynyldeoxyuridine (EdU) to label proliferating cells, and stained with antibodies to smooth muscle actin (SMA) and keratin‐8 (Krt8) (Figure 4F) (see materials and methods). The *Erbb3* ^*+/+;MMTV‐Cre*^ TEBs had a uniform SMA^+^ basal epithelial layer surrounding a mass of Krt8^+^ luminal epithelial cells. In contrast, the *Erbb3* ^*V943R/V943R;MMTV‐Cre*^ EdU^+^ TEBs harbored vacuoles between the basal and luminal layers (Figure 4F, arrowheads). The thickness of the basal and luminal layers was measured around the circumference of each TEB (Figure 4G). The basal layer was not significantly different in thickness between *Erbb3* ^*+/+;MMTV‐Cre*^ and *Erbb3* ^*V943R/V943R;MMTV‐Cre*^ TEBs (mean thickness of 7.18 mm ± 0.48 and 7.88 mm ± 0.29, respectively). However, the luminal layer was significantly thinner (mean thickness 23.24 mm ± 1.59 for *Erbb3* ^*+/+;MMTV‐Cre*^, 12.27 mm ± 0.87 for *Erbb3* ^*V943R/V943R;MMTV‐Cre*^), leading to a lower ratio of luminal to basal cell thickness in the *Erbb3* ^*V943R/V943R;MMTV‐Cre*^ TEBs.

### 2.5 A luminal epithelial subpopulation is reduced in number within Erbb3^V943R^ ^/V943R;MMTV‐Cre^ glands

A number of recent single cell studies of the mouse mammary gland^32^, ^33^, ^34^ have identified the major luminal and basal epithelial subsets that exist postnatally in nulliparous animals. Given the observed defect in ductal elongation in the *Erbb3* ^*V943R/V943R;MMTV‐Cre*^ mice, we interrogated the effect of the V943R mutation on mammary lumen cells using single cell RNA‐sequencing. Thoracic and abdominal mammary glands from homozygous 6‐week old *Erbb3* ^*+/+;MMTV‐Cre*^ and *Erbb3* ^*V943R/V943R;MMTV‐Cre*^ mice (n = 3 per genotype) were dissected, collagenase‐digested, pooled, enriched for epithelial populations and FACS sorted to obtain luminal cells.

**FIGURE 5:**
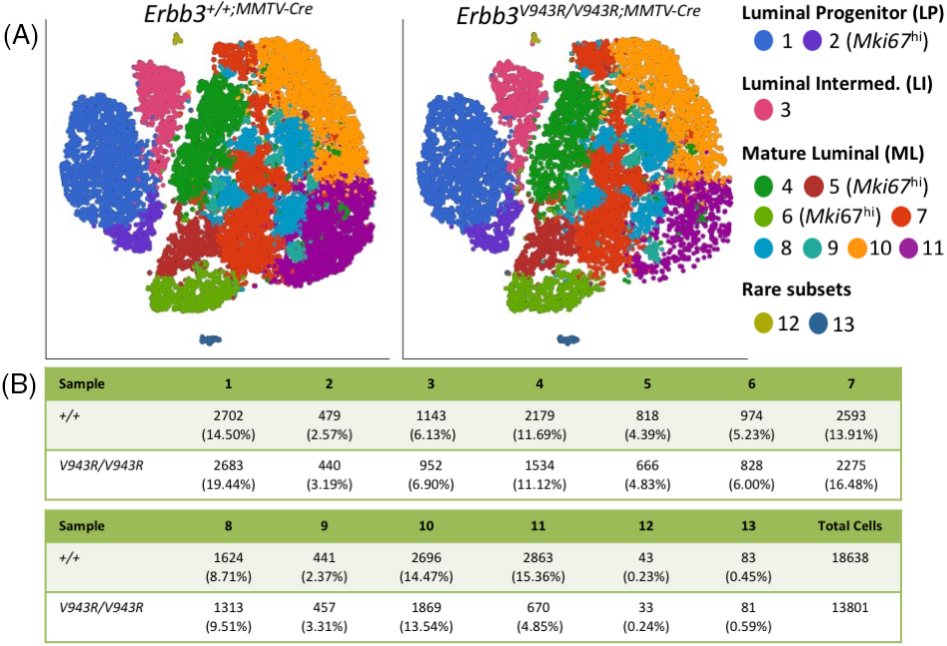
Single cell RNA‐sequencing analysis of luminal epithelial cells. A, Luminal epithelial cells were purified from the digested mammary glands of 6‐week old female mice and run on the 10× Chromium single cell RNA‐sequencing platform. Seurat tSNE plots were generated using Partek Flow (Partek) at a resolution of 0.6. LP, luminal progenitor; LI, luminal intermediate; ML, mature luminal. B, Summary of the numbers of cells within identified subclusters and their percentage as a function of total cell counts

A total of 18 638 and 13 801 cells from the homozygous *Erbb3* ^*+/+;MMTV‐Cre*^ and *Erbb3* ^*V943R/V943R;MMTV‐Cre*^ mice, respectively were analyzed (Figure 5). Thirteen distinct clusters were identified by Seurat tSNE analysis (Figure 5A). Overall, the cells showed near‐uniform expression of epithelial cell adhesion molecule (*Epcam*) and keratin‐18 (*Krt18*), with very little keratin‐5 (*Krt5*) expression (Figure S6A), confirming the identity of these cells as luminal epithelium (*Krt18* ^*+*^ *Krt5* ^*−*^) with very little to no basal epithelial (*Krt18* ^*−*^ *Krt5* ^*+*^) contamination. The cells could be subdivided into three major subpopulations (Figure S6B and C): a luminal progenitor (LP) population, characterized by *Kit* expression, a mature luminal (ML) population, defined by *Prlr* expression, and a luminal intermediate (LI) population, which expressed genes found in both the LP and ML populations in a graded fashion. This pattern is consistent with a previous study.^33^ In addition, subsets of proliferating cells were identified by *Mki67* expression (Figure S6B). *Erbb2* and *Erbb3* mRNA expression was detected within the major subpopulations (Figure S6C and D), with *Erbb3* mRNA showing a ~1.4‐fold decrease in the mutant sample.

Further (Figure 5A), the LP population was divisible into two subpopulations (clusters 1 and 2), one of which was distinguished by elevated *Mki67* expression (cluster 2), while the LI cell population (cluster 3) was not further subdivided by the tSNE analysis. The ML population was divided into eight subpopulations (clusters 4 through 11), with two showing elevated *Mki67* expression relative to the other populations (clusters 5 and 6). Two rare populations were also identified (clusters 12 and 13). Most of the subpopulations existed at a similar percentage of total cell numbers between *Erbb3* ^*+/+;MMTV‐Cre*^ and *Erbb3* ^*V943R/V943R;MMTV‐Cre*^ genotypes, with a few exceptions (Figure 5B). Clusters 1 and 7 were slightly elevated in the mutant sample, increasing by ~5% and ~2.6% relative to the conditional knock‐in mice, respectively. Cluster 11 showed the most striking difference between genotypes, dropping from 15.36% of the total cell number in the *Erbb3* ^*+/+;MMTV‐Cre*^ sample to 4.85% of the total cell number in the *Erbb3* ^*V943R/V943R;MMTV‐Cre*^ sample, a more than 3‐fold reduction. The loss of this population in the mutant sample suggests that ERBB3 activator function positively regulates the differentiation, proliferation, or survival of a subset of mature luminal epithelial cells.

**FIGURE 6:**
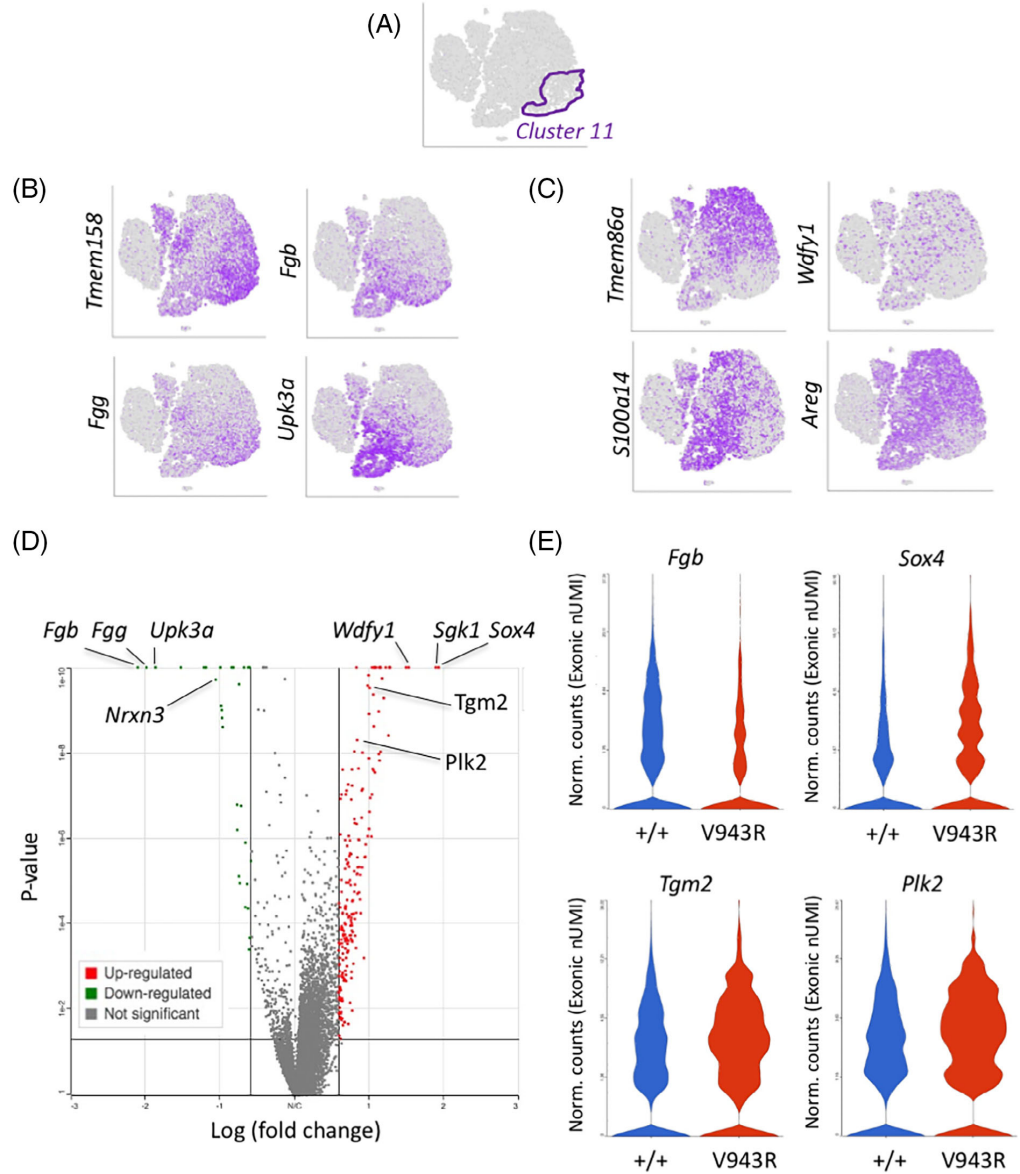
Differential gene expression analysis of subcluster 11. A, Outline of cluster 11 cells. B, Representative mRNAs differentially expressed at higher levels in *Erbb3* ^*+/+;MMTV‐Cre*^ subcluster 11 than in other mature luminal subclusters. C, Representative mRNAs differentially expressed at lower levels in *Erbb3* ^*+/+;MMTV‐Cre*^ subcluster 11 than in other mature luminal subclusters. D, Volcano plot of genes within subcluster 11 that show at least 1.5‐fold higher or lower mRNA expression in the *Erbb3* ^*V943R/V943R;MMTV‐Cre*^ sample relative to *Erbb3* ^*+/+;MMTV‐Cre*^. E, Transcript expression level of select mRNAs *Fgb*, *Sox4*, *Tgm2*, and *Plk2*

To further understand the cells in cluster 11 we performed transcript enrichment analysis (Figure 6A‐C). The transcripts elevated within cluster 11 *Erbb3* ^*+/+;MMTV‐Cre*^ cells include transmembrane protein 158 (*Tmem158*), fibrinogens‐γ and ‐β (*Fgg* and *Fgb*), and Uroplakin‐3a (*Upk3a*) (Figure 6B). Conversely, genes showing lower transcript levels within cluster 11 *Erbb3* ^*+/+;MMTV‐Cre*^ cells compared to the other identified clusters included transmembrane protein 86a (*Tmem86a*), S100 calcium‐binding protein a14 (*S100a14*), WD repeat and FYVE domain‐containing protein 1 (*Wdfy1*), and the EGFR ligand amphiregulin (*Areg*) (Figure 6C). This signature suggests that cluster 11 cells might regulate epithelial‐matrix interactions during mammary development.

To determine which genes changed in expression within cluster 11 between *Erbb3* ^*+/+;MMTV‐Cre*^ and *Erbb3* ^*V943R/V943R;MMTV‐Cre*^ genotypes, we performed differential expression (DE) analysis on cluster 11 (Figure 6D,E). We found 30 genes that showed a 1.5‐fold or greater reduction in transcript levels in *Erbb3* ^*V943R/V943R;MMTV‐Cre*^ relative to *Erbb3* ^*+/+;MMTV‐Cre*^ cluster 11 cells, while 206 genes showed 1.5‐fold or more elevated transcript levels in *Erbb3* ^*V943R/V943R;MMTV‐Cre*^ relative to *Erbb3* ^*+/+;MMTV‐Cre*^ cluster 11 cells. The transcripts for *Fgg*, *Fgb*, and *Upk3a* that were enriched within cluster 11 from *Erbb3* ^*+/+;MMTV‐Cre*^ mice are also downregulated on a per‐cell basis within *Erbb3* ^*V943R/V943R;MMTV‐Cre*^ glands. Downregulation of fibrinogens‐γ and ‐β suggests a positive role for the ERBB3 receptor in extracellular matrix (ECM) production. *Upk3a* is a single‐pass transmembrane protein that forms complexes on the apical side of urinary bladder epithelial cells,^35^ that was recently shown to mark a distinct population of epithelial progenitors in the lung that contribute to post‐injury repair.^36^ Genes showing increased transcript levels in the mutant sample include the Sex‐determining region Y‐related high‐mobility‐group transcription factor 4 (*Sox4*), Tissue transglutaminase 2 (*Tgm2*), and Polo‐like kinase 2 (*Plk2*). SOX4 plays a role as a tumor suppressor or oncogene, depending on cellular context.^37^ TGM2 can be localized to both the cytosol and to extracellular spaces, where it mediates the covalent cross‐linking of several proteins. Intracellular TGM2 is thought to play an important role in apoptosis, while extracellular TGM2 is involved in cell adhesion, ECM stabilization, cell proliferation, and motility.^38^ PLK2 is a serine/threonine kinase that may slow mitotic progression in response to stress signaling.^39^

### 2.6 Residual kinase activity of ErbB3 is dispensable for normal development

**FIGURE 7:**
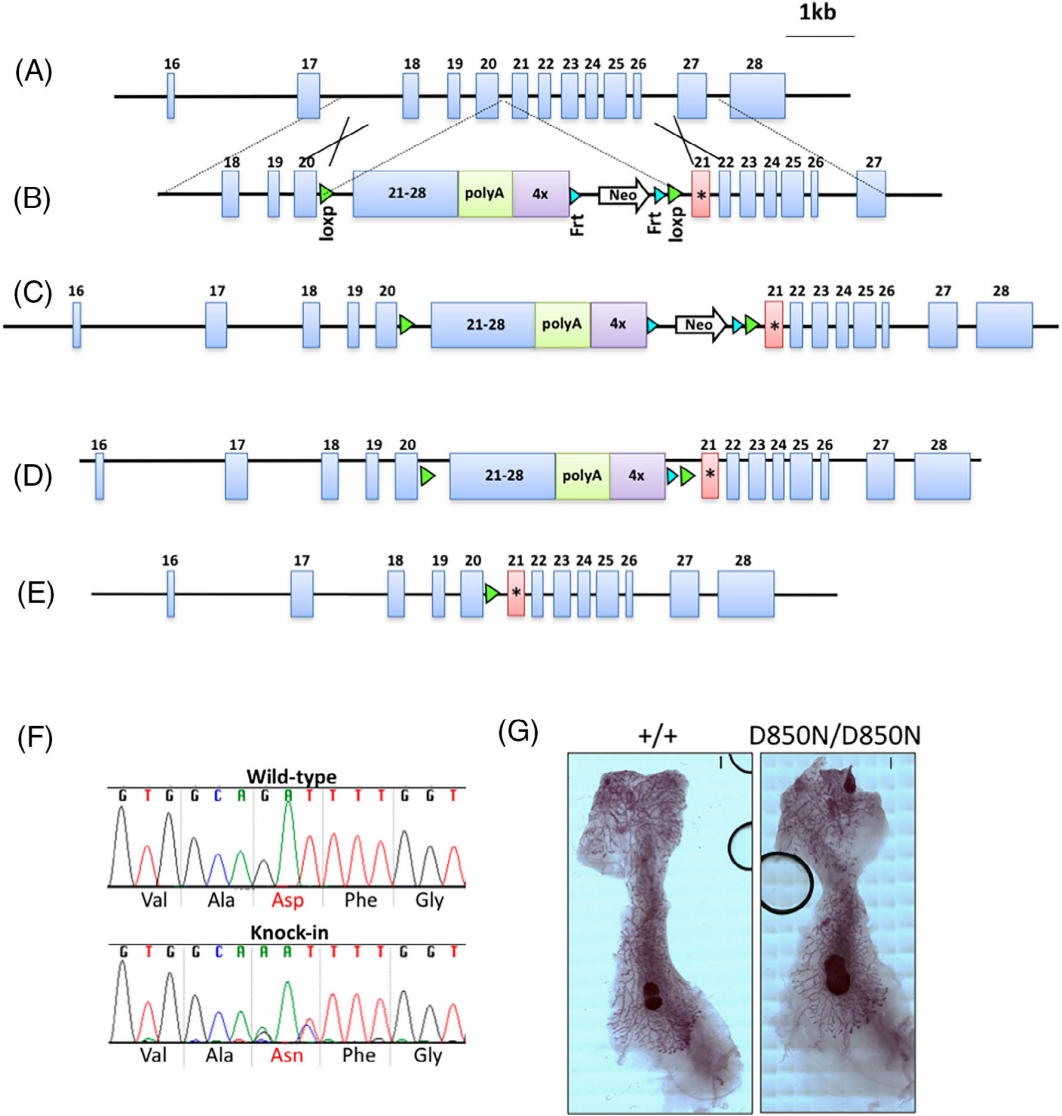
Generation of mice with a conditional knock‐in *Erbb3* ^*D850N*^ allele. A, Genomic region of the *Erbb3* gene. B, Targeting vector used to modify the *Erbb3* locus. Homology arms for targeting of ES cells are indicated. An asterisk marks the location of the D850N mutation. C, Targeted *Erbb3* locus with Neo selection marker present. D, *Erbb3* ^*CKI‐D850N*^ conditional knock‐in allele after FLP‐mediated excision of Neo. E, *Erbb3* ^*D850N*^ knock‐in allele after Cre‐mediated excision of the floxed wildtype *Erbb3* cDNA. F, RT‐PCR validation of mouse genotypes. RNA isolated from wild‐type or *Erbb3* ^*D850N*^ knock‐in ES cells following Cre mediated activation was converted to cDNA and amplified using gene‐specific primers flanking the region of interest confirms the expression of D850N allele. G, Images of representative mammary glands from *Erbb3* ^*+/+;MMTV‐Cre*^ and *Erbb3* ^*D850N/D850N;MMTV‐Cre*^ animals. Scale bar = 1 mm

To study the relevance of the residual kinase activity of ERBB3 *in vivo*, we engineered a mouse capable of conditionally expressing a kinase‐dead *Erbb3* ^*D850N*^ allele under its native promoter (Figure 7 and Figure S1). In this genetically engineered mouse model we replaced the WT exon 21 (Figure 7A) in embryonic stem cells with a targeting vector composed of a *LoxP* flanked cDNA minicassette coding for exon 21‐28, a polyA signal sequence and a transcriptional stop cassette (Figure 7B). The modified wild‐type exon 21 was followed by a copy of *Erbb3* exon 21 encoding the D850N mutation (Figure 7C). Embryonic stem cells with the conditional knock‐in (CKI) allele *Erbb3* ^*CKI‐V943R*^ were created following FLP‐mediated excision of Neo (Figure 7D). To induce whole animal expression of the mutant D850N allele developing *Erbb3* ^*CKI‐D850N/+*^ embryos were treated with the cell‐permeant HTN‐Cre recombinase at E0.5 (Figure 7E). Intercrosses between *Erbb3* ^*CKI‐D850N/+*^; HTN‐Cre mice resulted in viable homozygous *Erbb3* ^*D850N/D850N*^ animals that developed normally. Knock‐in was confirmed by RT‐PCR and sequencing (Figure 7F). Further, conditional activation of D850N expression in mammary glands (*Erbb3* ^*D850N/D850N;MMTV‐Cre*^) did not lead to defects in mammary duct development (Figure 7G). These data together indicate that the residual kinase activity of ERBB3 is dispensable for normal development.

## 3 DISCUSSION

The ERBB family of receptors plays an important role in developmental and cancer.^4^ Overlapping and distinct phenotypes result from the loss of each ERBB receptor, resulting from differences in amino acid sequences, expression patterns, proteolytic cleavage events, dimer composition, and ligand utilization among the family members. Mice lacking the genes for *Neuregulin‐1* (*Nrg1*), *Erbb2*, *Erbb3*, and *Erbb4* display an overlapping set of defects, with the phenotype resulting from the loss of *Nrg1* encompassing aspects of the other three *Erbb* receptor knockout phenotypes. During development of the heart, for example, critical ERBB‐mediated signals are exchanged between the endocardium and the opposing myocardium around E9.0. The endocardium emits soluble NRG1 protein, while the neighboring myocardial cells express ERBB2 and ERBB4.^40^ NRG binding to ERBB4/4 homodimers or ERBB2/4 heterodimers in the myocardium drives the proliferation of cardiac myocytes, which form finger‐like projections of heart muscle known as trabeculae that are essential for efficient cardiac contraction and embryonic blood circulation. *Nrg1* null mice die mid‐gestation (E10.5) from cardiac failure due to the poor development of these ventricular trabeculae, as well as endocardial cushion defects.^18^, ^41^, ^42^ As expected, given their expression pattern, an identical defect in trabeculae formation is seen in mice null for *Erbb2*,^18^, ^43^, ^44^ and *Erbb4*.^45^

ERBB3 is expressed by the endocardial cushion mesenchyme that separates embryonic atrium and ventricle and later gives rise to the heart valves.^18^ This tissue becomes noticeably thinner in *Erbb3* ^*−/−*^ embryos. As a result, the heart valves fail to completely develop, leading to embryonic death around E13.5. Unlike *Erbb2* or *Erbb4* embryonic knockout, trabeculation is marginally impacted in *Erbb3* ^*−/−*^ embryos, although a slight thinning of the myocardium has been noted.^18^

In this study, we tested the relevance of the “activator” function of ERBB3 by engineering a V943R activator‐function impaired knock‐in mutant mice. Homozygous expression of the mutant V943R allele led to embryonic lethality at E12.0 with heart defects similar to those observed in *Erbb3* ^*−/−*^ embryos.^18^ The ventricular walls were thinner than expected by E10.5, which is consistent with the myocardial thinning reported in *Erbb3* ^−/−^ embryos.^18^ In addition, a marked reduction in the thickness of the endocardial cushion was seen in the *Erbb3* ^*V943R/V943R*^ embryos by E11.5, as would be expected if the cushion mesenchymal cells were less proliferative. These similarities between *Erbb3* ^−/−^ and *Erbb3* ^*V943R/V943R*^ embryos suggest that the V943R point mutation results in a complete loss of ERBB3 receptor function during embryonic heart development. Unlike the *Erbb3* ^−/−^ knock‐out mice where ERBB3 protein is lost, the *Erbb3* ^*V943R/V943R*^ knock‐in preserves the ERBB3 protein, and yet resulted in embryonic lethality. This showed that the activator function of ERBB3 over its scaffolding role is critical for signaling during development. Defects in neural development and other organs such as stomach, pancreas and adrenal gland development have been reported in *Erbb3* ^*−/−*^ null mice.^18^, ^19^ Our study did not examine the *Erbb3* ^*V943R/V943R*^ embryos for these additional defects but focused on ERBB3 activator function in mammary glands as conditional activation of V943R can eventually be used to test the role of ERBB3 in ERBB2‐driven mammary tumorigenesis.

During development and pregnancy, each member of the ERBB family controls facets of mammary gland architecture and function. The ERBB4 receptor is essential for lactation through regulation of STAT5 but is dispensable for duct development despite being detected in the epithelial compartment.^46^, ^47^, ^48^ EGFR is expressed both by epithelial cells within TEBs and by the surrounding stroma, but it is only stromal EGFR that is required for ductal growth during puberty.^49^ ERBB2 and ERBB3 are expressed in the epithelial cells of the developing ductal network of the glands during puberty and are both essential for the development of this compartment. Transplanting *Erbb2‐* or *Erbb3*‐null mammary buds from embryos into cleared or de‐epithelialized^50^ WT recipient mice results in defective ductal outgrowth during puberty,^22^, ^51^ indicating an epithelial‐intrinsic requirement for both receptors. Targeted ablation of *Erbb2* or *Erbb3* by MMTV‐directed Cre‐mediated recombination results in a similar shortened duct phenotype.^23^, ^52^ Detailed cellular analysis shows that loss of *Erbb3* in the mammary epithelium results in disorganized TEBs^23^ with decreased body cell proliferation as measured by Ki67 IHC staining as well as formation of vacuoles between basal and luminal layers. The outcome of knocking in *Erbb3* bearing the V943R mutation in the mammary luminal epithelium is very similar, suggesting ERBB3 functions as a dedicated activator in certain subpopulations of mammary duct epithelial cells.

We used single cell RNA‐sequencing to assess the mRNA expression changes in freshly isolated luminal epithelial cells. A subcluster of ML epithelial cells that comprise ~15% of the total luminal epithelial cell number in the WT sample was reduced by more than 3‐fold (to ~4.9%) in the *Erbb3* ^*V943R/V943R;MMTV‐Cre*^ sample. Surprisingly, this subcluster was not among the more proliferative subclusters as marked by *Mki67* mRNA expression. Relative to other ML subclusters in the WT sample, this subcluster showed elevated transcript levels of fibrinogens γ and β, suggesting a role in epithelial‐matrix interactions. Moreover, a reduction in transcripts for these genes was observed in the *Erbb3* ^*V943R/V943R;MMTV‐Cre*^ background, demonstrating a requirement for ERBB3 receptor signaling function for their production. Fibrinogen is one of many extracellular matrix (ECM) molecules such as collagen, elastin, and laminin that facilitate both normal and cancer cell migration, and regulate intercellular communication through growth factor sequestration.^53^ Fibrinogen expression has been observed in alveolar epithelial cells,^54^ the human breast cancer line MCF‐7,^53^ and several carcinomas,^55^, ^56^ and has also been documented at the tumor‐host cell interface.^57^ The reduction in the fibrinogen‐expressing luminal cell subpopulation in the *Erbb3* ^*V943R/V943R;MMTV‐Cre*^ mice, combined with the reduction in transcript levels per cell as seen in the differential expression analysis, could be indicative of a substantial change in ECM composition within the *Erbb3* ^*V943R/V943R;MMTV‐Cre*^ mammary gland, which may influence duct elongation rates.

Another transcript expressed by the V943R‐dependent luminal subcluster encoded uroplakin UPK3A. Uroplakins are single‐pass transmembrane proteins abundant in the urinary bladder epithelium, or uroepithelium, where they form structures in the outer leaflet of the cell membrane. These structures decrease cell permeability to ions, substances found in urine, and bacteria.^58^ More recently, UPK3A was determined to be a marker of rare, specialized club cells (U‐CCs) in the lung epithelium that regenerate airways in response to damaging agents such as naphthalene.^36^ Whether the cells expressing *Upk3a* in the mammary duct epithelium have a similar regenerative potential remains an intriguing question.

How ERBB3 is integrated with the complex hormone‐ and growth factor‐driven morphogenetic programs of the mammary gland remains an open question. Numerous autocrine and paracrine signaling loops among epithelial cells, as well as reciprocal stromal‐epithelial interactions, play a critical role in development of the glands. Factors such as insulin‐like growth factor 1 (IGF1), hepatocyte growth factor (HGF), epidermal growth factor (EGF), and fibroblast growth factor (FGF) induce cell proliferation, survival, and branching.^59^ The ECM also has an important role to play by providing a substrate for cell adhesion and migration, facilitating mechanosensing, promoting angiogenesis, and sequestering growth factors.^53^, ^60^ Insights from ERBB3's role during mammary development can have implications for our understanding of tumor biology in the context of ERBB2/ERBB3 oncogenic mutation and/or amplification. It is important to remember that while *Erbb3* ^*V943R*^ shares many aspects of the reported *Erbb3‐KO* phenotype, subtle differences may exist that could indicate additional scaffolding functions for the ERBB3 receptor.

Unlike *Erbb3* ^*V943R/V943R*^ mice, we found that mice bearing D850N mutation (*Erbb3* ^*D850N/D850N*^) in the canonical “DFG” motif of the ERBB3 kinase domain, that is expected to abolish any residual kinase activity developed normally. Consistent with our findings, a recent study reported normal development of a kinase‐dead ERBB3 K740M variant knock‐in mouse.^61^ These findings, together with the functional defects observed for the ERBB3 V943R mutant, indicate that ERBB3 primarily functions as an allosteric activator during tissue morphogenesis, and that its reported weak kinase activity are dispensable for its key physiological function. Our *Erbb3* ^*CKI‐V943R*^ mice will be an important tool to test and clarify the relevance of ERBB3 in ERBB2 driven tumorigenesis and drug targeting.

## 4 MATERIALS AND METHODS

### 4.1 Plasmids and antibodies

Human ERBB2 bearing an N‐terminal herpes simplex glycoprotein D (gD) tag was expressed using the pLPCX retroviral vector (Clontech). N‐terminally Flag‐tagged wild‐type and mutant ERBB3 were expressed using retroviral vector pRetro‐IRES‐GFP.^31^ Antibodies recognizing pERBB2 (Y1221/22) (Cat No. 2249), pERK1/2 (Cat No. 9101), total ERK (Cat No. 4696), ERBB2 (Cat No. 2242) and β‐Actin (Cat No. 4967) were obtained from Cell Signaling Technology.

### 4.2 Ba/F3 assays

Ba/F3 pro‐B cells (DSMZ, Germany) were washed with PBS and resuspended in media (RPMI +10% FBS) without IL‐3, then plated at 10 000 cells/well in 96‐well plates. On day 0 and day 4 cell viability was measured using Cell Titer‐Glo Luminescence Cell Viability kit (Promega) and plates were read on a Synergy 2 luminescence plate reader (Biotek Instruments). The luminescence reading on day 4 divided by the reading on day 0 was used to express relative cell survival.

### 4.3 Generation of Erbb3 V943R conditional (inducible) knock‐in mice

Mice carrying the *Erbb3* ^*CKI‐V943R*^ allele were generated using C57BL/6N ES cells as previously described.^62^ Fertilized, one‐celled embryos (zygotes) were microinjected on E0.5. Briefly, the *Erbb3* ^*CKI‐V943R*^ allele was obtained by inserting a cassette containing a *LoxP*, wild‐type *Erbb3* cDNA (exons 23‐28), a human growth hormone 3′UTR followed by a 4x polyadenylation (polyA) signal, an *FRT‐Neo* ^*R*^ *‐FRT* selection marker and a second *LoxP*, into the *Erbb3* locus 178 bp 5′ of a mutated exon 23 encoding the V943R (GTC to AGG) change. The *Neo* ^*R*^ cassette was excised in ES cells using adeno‐FLP prior to microinjection. *Erbb3* ^*CKI‐V943R/+*^ mice were maintained on a C57BL/6N genetic background. A constitutive *Erbb3* ^*V943R*^ knock‐in allele was generated by treating *Erbb3* ^*CKI‐V943R/+*^ heterozygous zygotes with His‐tagged, nuclear localization signal (NLS)‐bearing TAT‐Cre recombinase (HTN‐Cre), essentially as described.^63^ Mammary tissue‐specific *Erbb3* ^*V943R*^ knock‐in was achieved by intercrossing mice carrying *Erbb3* ^*CKI‐V943R*^ and *MMTV‐Cre* alleles. Knock‐in was confirmed by RT‐PCR and sequencing. Briefly, RNA was extracted from kidney in the case of *Erbb3* ^*V943R/+*^ mice, or from purified mammary epithelial cells in the case of *Erbb3* ^*V943R/V943R;MMTV‐Cre*^ mice using an RNeasy Kit (Qiagen). cDNA was synthesized by cDNA Reverse Transcription kit (Stratagene), and PCR‐amplified with the following primers: 5′‐TGCAGCTTGTCACTCAGTACTTGCCTCTG‐3′ and 5′‐TGGTGCTCAGAGCAGATGGCTCTGCTG‐3′. The PCR product was purified by QIAquick PCR Purification Kit (Qiagen) and sequenced using primer 5′‐ATGGTGCATAGGGACCTTG‐3′.

### 4.4 Generation of Erbb3 D850N conditional (inducible) knock‐in mice

Using the same strategy as done with V943R knock‐in mice we also generated mice bearing a D850N mutation in the canonical DFG motif of the ERBB3 kinase domain. The D850N mutation is expected to abolish any residual kinase activity in the pseudokinase domain of ERBB3.^11^, ^12^, ^64^ The expression of D850N allele was confirmed (Figure 7F) following HTN‐Cre‐mediated activation in ES cells using PCR. Primers used were same as those used to confirm V943R allele, except that we used 5′‐TCTCGTCAATCATCCAACAC‐3′ and 5′‐GACCATGACCATGTAGACGTC‐3′ for sequencing. We activated the expression of *Erbb3* D850N allele both during embryonic development (*Erbb3* ^*D850N/D850N*^) and in developing mammary glands using the *MMTV‐Cre* driver (*Erbb3* ^*D850N/D850N;MMTV‐Cre*^).

### 4.5 Embryonic phenotyping

Embryonic mice with the genotype *Erbb3* ^*V943R/V943R*^ (C57BL/6N genetic background, mouse strain designation 008423) as well as developmental age‐matched *Erbb3* ^*V943R/+*^ controls were harvested at E10.5, E11.5, E12.5, and E13.5, where the morning on which a copulation plug was observed was designated as E0.5. The mating period was overnight for conventional matings but limited to a 2 hours window at the end of the dark period for timed mattings. Embryos were fixed in neutral buffered 10% formalin, processed conventionally into paraffin, and sectioned serially in the sagittal orientation at 4 μm. For each embryo, every 10th section was stained with hematoxylin and eosin and assessed histopathologically.

### 4.6 Mammary gland carmine staining

Abdominal mammary glands were mounted onto glass slides, briefly dried, and then fixed overnight in Carnoy's fixative (60% ethanol, 30% cholorform, 10% glacial acetic acid). Glands were gradually rehydrated over the course of a day and then stained overnight in carmine alum solution (Stem Cell Technologies). Glands were destained in 50% ethanol, 0.25 N HCl for 4 hours or until sufficient contrast between the epithelium and fat pad was observed. Samples were then dehydrated for at least one hour each in 70%, 95%, and 100% ethanol before clearing in HistoClear solution (National Diagnostics) overnight.

### 4.7 Mammary gland morphological analysis

Whole mounts were imaged as a collection of tiled images using a 4× objective on a Nikon Ti‐E microscope with Fi1 color camera (Nikon Instruments). To get the entire duct network in focus, five focal planes were captured and compiled into a 2D image using Extended Depth of Focus software (Nikon Instruments). To quantify total duct elongation the length from the center of the lymph node to the furthest end of the ducts along the main axis of the gland was measured using Nikon Elements software (Nikon Instruments). Color images were converted into greyscale images by removing 2 color channels and inverting to create a monochrome image in Imaris software (Bitplane). Percent area and branching measurements were made by creating a binary mask of the ducts using the Surfaces function in Imaris and applying a gaussian filter. The resulting images were imported into ImageJ, skeletonized, and then analyzed using the Analyze Skeleton 2D/3D plugin (ImageJ).

### 4.8 Processing and imaging of whole mount mammary glands

Mice were injected intraperitoneally with 100 mg/kg of ethynyldeoxyuridine (EdU) to label proliferating cells. Animals were deeply anesthetized using isoflurane and perfused with PBS containing heparin followed by tissue fixation using 4% paraformaldehyde (PFA), as described previously.^65^ The tissue was harvested and subjected to immunolabeling using a previously published staining protocol that avoided methanol treatment steps.^66^ Antibodies to smooth muscle actin (SMA) and keratin‐8 (KRT8) were used. EdU^+^ TEBs were identified by AF647 labeling using the Click‐IT Plus kit (Thermo Fisher Scientific). Glands were cleared using the FluoClearBABB method,^67^ and high‐resolution 3‐dimensional images acquired using a Leica SP8 microscope equipped with a white light laser and Leica BABB immersion lenses (HCX PL FLUOTAR 5×/0.15 IMM lens for low‐resolution and HCX APO L 20×/0.95 IMM lens for high resolution images). Imaging data was analyzed on a power workstation using Imaris software (Bitplane).

### 4.9 Flow cytometry

Ba/F3 cells stably expressing empty vector or expression vectors for ERBB2 and ERBB3 were stained with PE‐conjugated antibodies targeting each receptor (BD Biosciences) for 20 minutes in the presence of a human FcR‐blocking reagent (Miltenyi), washed, and then read on a FACS Calibur (BD Biosciences). For mammary gland analysis, pairs of abdominal mammary glands from 6‐week old mice were digested overnight in gentle collagenase (Stem Cell Technologies) per manufacturer's instructions, followed by digestion with trypsin and DNAse I. Cells were stained with an antibody cocktail containing propidium iodide, anti‐mouse CD16/CD32 (clone 2.4G2, BD Biosciences), anti‐CD24 (BD Biosciences), and anti‐CD45, ‐CD31, ‐Ter119, ‐CD29, ‐CD49f, and ‐EpCAM (BioLegend).

### 4.10 Single cell isolation, sequencing, and analysis

The thoracic and abdominal mammary glands from 6‐week old *Erbb3* ^*+/+;MMTV‐Cre*^ or *Erbb3* ^*V943R/V943R;MMTV‐Cre*^ mice (N = 3 per group) were pooled, minced, and collagenase‐digested (Stem Cell Technologies) with constant agitation for 1 hour. The samples were further dissociated using an AutoMACS, followed by treatment with Trypsin‐EDTA and DNAse I (StemCell Technologies) according to the manufacturer's recommendations. Epithelial populations were enriched using the EasySep mouse epithelial cell enrichment kit II (StemCell Technologies), and then FACS sorted for luminal epithelial populations as shown in Figure S5. Post‐sorting, the density and viability of the single‐cell suspensions was determined by Vi‐CELL XR cell counter (Beckman Coulter), and the cell density was then used to impute the volume of single cell suspension needed in the reverse transcription master mix, aiming to achieve ~6000 cells per sample. The single‐cell suspension was then processed for single‐cell RNA‐seq analysis using the Chromium Single Cell 3′ Library and Gel Bead Kit v2 (10× Genomics). cDNAs and libraries were prepared as described per the manufacturer's instructions (10× Genomics). Libraries were profiled by Bioanalyzer High Sensitivity DNA Kit (Agilent Technologies) and quantified using the Kapa Library Quantification Kit (Kapa Biosystems). Each library was sequenced in one lane of HiSeq4000 (Illumina) following the manufacturer's sequencing specification (10× Genomics). Data was analyzed using the Partek Flow program (Partek) with a Seurat setting of 0.6.

### 4.11 Animal studies

All animal studies were conducted under protocols approved in advance by Genentech's Institutional Animal Care and Use Committee (IACUC) guidelines. Animals were housed in filter‐capped polycarbonate cages (6 per cage for adults, singly for dams with offspring), fed pelleted chow and purified water ad libitum, and were maintained at constant environmental conditions (12 hr light‐dark cycle, 22 ± 2°C).

### 4.12 Statistical analyses

Prism (GraphPad Software) was used for statistical analyses. The number of biological replicates is denoted by the sample number (N). The Student's *t* test was used for the evaluation of significance as specified in the figure legends. n.s. = not significant (*P* > .05), *P < .05, **P < .01, ***P < .001.

## AUTHOR CONTRIBUTIONS

**Somasekar Seshagiri:** Conceptualization; formal analysis; investigation; methodology; project administration; supervision; writing‐original draft; writing‐review and editing. **Kate Senger:** Formal analysis; investigation; methodology; writing‐review and editing. **Wenlin Yuan:** Investigation. **Meredith Sagolla:** Investigation. **Jonas Doerr:** Investigation. **Brad Bolon:** Investigation. **James Ziai:** Investigation. **Kai‐Hui Sun:** Investigation. **Soren Warming:** Investigation. **Merone Roose‐Girma:** Investigation. **Na Zhang:** Investigation. **Lucinda Tam:** Investigation. **Robert Newman:** Investigation. **Subhra Chaudhuri:** Investigation. **Aju Antony:** Investigation. **Leonard Goldstein:** Investigation. **Steffen Durinck:** Investigation. **Bijay Jaiswal:** Investigation. **Daniel Lafkas:** Investigation. **Zora Modrusan:** Investigation.

## CONFLICT OF INTEREST

The authors from Genentech hold company shares/options.

### DATA AVAILABILITY STATEMENT

Expression data has been submitted to Gene Expression Omnibus under accession # GSE146437.

### PEER REVIEW

The peer review history for this article is available at https://publons.com/publon/10.1002/ggn2.10036.

## Supporting information


**Appendix** **S1**: Supporting information

**Appendix** **S2**: Supporting information
