# Supplementary material for: Embryonic lethality and defective mammary gland development of activator‐function impaired conditional knock‐in Erbb3 V943R mice
Source: Adv Genet (Hoboken). 2020 Dec 5;2(1):e10036. doi: 10.1002/ggn2.10036 (PMC9744554; doi:10.1002/ggn2.10036)
Supplement: Supplementary file 1 — Appendix S1: Supporting information [file GGN2-2-e10036-s002.pdf]

Table S1. Expected and observed genotypes of live offspring following systemic expression of ERBB3 V943R (*Erbb3*$^{V943R/V943R}$).

Genotype of live offspring (*Erbb3*$^{CKI-V943R/+}$; HTN-Cre x *Erbb3*$^{CKI-V943R/+}$; HTN-Cre)			
Erbb3	Observed Number of Pups (n=211)	Observed Ratio	Expected Ratio
+/+	80	38%	25%
V943R/+	131	62%	50%
V943R/V943R	0	0%	25%

Table S2. Expected and observed genotypes of live offspring following mammary-specific expression of ERBB3 V943R (*Erbb3*$^{V943R/V943R;MMTV\text{-}Cre}$).

Genotype of live offspring (*Erbb3*$^{CKI-V943R/+}$ x *Erbb3*$^{CKI-V943R/+}$;*MMTV-Cre*)				
Erbb3	*MMTV-Cre*	Observed Number of Pups (n=420)	Observed Ratio	Expected Ratio
+/+	pos	68	16.2%	12.5%
+/+	neg	48	11.4%	12.5%
V943R/+	pos	128	30.5%	25%
V943R/+	neg	98	23.3%	25%
V943R/V943R	pos	48	11.4%	12.5%
V943R/V943R	neg	30	7.1%	12.5%

```
                            P-loop
h-ERBB3      ARIFKETELRKLKVLGSGVFGTVHKGVWIPEGESIKIPVCIKVIEDKSGRQSFQAVTDHM   760
m-ERBB3      ARIFKETELRKLKVLGSGVFGTVHKGIWIPEGESIKIPVCIKVIEDKSGRQSFQAVTDHM   758
h-ERBB4      LRILKETELKRVKVLGSGAFGTVYKGIWVPEGETVKIPVAIKILNETTGPKANVEFMDEA   769
h-EGFR       LRILKETEFKKIKVLGSGAFGTVYKGLWIPEGEKVKIPVAIKELREATSPKANKEILDEA   763
h-ERBB2      MRILKETELRKVKVLGSGAFGTVYKGIWIPDGENVKIPVAIKVLRENTSPKANKEILDEA   771
                            GxGxxG                 VAIK

h-ERBB3      LAIGSLDHAHIVRLLGLCPGSSLQLVTQYLPLGSLLDHVRQHRGALGPQLLLNWGVQIAK   820
m-ERBB3      LAVGSLDHAHIVRLLGLCPGSSLQLVTQYLPLGSLLDHVRQHRETLGPQLLLNWGVQIAK   818
h-ERBB4      LIMASMDHPHLVRLLGVCLSPTIQLVTQLMPHGCLLEYVHEHKDNIGSQLLLNWCVQIAK   829
h-EGFR       YVMASVDNPHVCRLLGICLTSTVQLITQLMPFGCLLDYVREHKDNIGSQYLLNWCVQIAK   823
h-ERBB2      YVMAGVGSPYVSRLLGICLTSTVQLVTQLMPYGCLLDHVRENRGRLGSQDLLNWCMQIAK   831

                                            D850N
                                            |  ___activation loop___
h-ERBB3      GMYYLEEHGMVHRNLAARNVLLKSPSQVQVADFGVADLLPPDDKQLLYSEAKTPIKWMAL   880
m-ERBB3      GMYYLEEHSMVHRDLALRNVMLKSPSQVQVADFGVADLLPPDDKQLLHSEAKTPIKWMAL   878
h-ERBB4      GMMYLEERRLVHRDLAARNVLVKSPNHVKITDFGLARLLEGDEKEYNADGGKMPIKWMAL   889
h-EGFR       GMNYLEDRRLVHRDLAARNVLVKTPQHVKITDFGLAKLLGAEEKEYHAEGGKVPIKWMAL   883
h-ERBB2      GMSYLEDVRLVHRDLAARNVLVKSPNHVKITDFGLARLLDIDETEYHADGGKVPIKWMAL   891
                        HRD                 DFG

h-ERBB3      ESIHFGKYTHQSDVWSYGVTVWELMTFGAEPYAGLRLAEVPDLLEKGERLAQPQICTIDV   940
m-ERBB3      ESIHFGKYTHQSDVWSYGVTVWELMTFGAEPYAGLRLAEIPDLLEKGERLAQPQICTIDV   938
h-ERBB4      ECIHYRKFTHQSDVWSYGVTIWELMTFGGKPYDGIPTREIPDLLEKGERLPQPPICTIDV   949
h-EGFR       ESILHRIYTHQSDVWSYGVTVWELMTFGSKPYDGIPASEISSILEKGERLPQPPICTIDV   943
h-ERBB2      ESILRRRFTHQSDVWSYGVTVWELMTFGAKPYDGIPAREIPDLLEKGERLPQPPICTIDV   951

                 V943R
                 |
h-ERBB3      YMVMVKCWMIDENIRPTFKELANEFTRMARDPPRYLVIK---------------------   979
m-ERBB3      YMVMVKCWMIDENIRPTFKELANEFTRMARDPPRYLVIK---------------------   997
h-ERBB4      YMVMVKCWMIDADSRPKFKELAAEFSRMARDPQRYLVIQGDDRMKLPSPNDSKFFQNLLD   1009
h-EGFR       YMIMVKCWMIDADSRPKFRELIIEFSKMARDPQRYLVIQGDERMHLPSPTDSNFYRALMD   1003
h-ERBB2      YMIMVKCWMIDSECRPRFRELVSEFSRMARDPQRFVVIQ---------------------   990

h-ERBB3      -------------        ---
m-ERBB3      -------------        ---
h-ERBB4      EE-----------        1011
h-EGFR       EEDMDDVVDADEY        1016
h-ERBB2      -------------        ---
```

Figure S1. Multiple sequence alignment of ERBB receptor kinase domains. Conserved elements are shaded in grey and mutated sites are highlighted in yellow. Protein sequences used in the alignment are as follow: h-ERBB3 NP_001973: 701..979, m-ERBB3 NP_034283: 699 ..977; h-ERBB4 NP_005226: 710..1012, h-EGFR NP_005219: 704-1016, and h-ERBB2 NP_004439: 712..990.

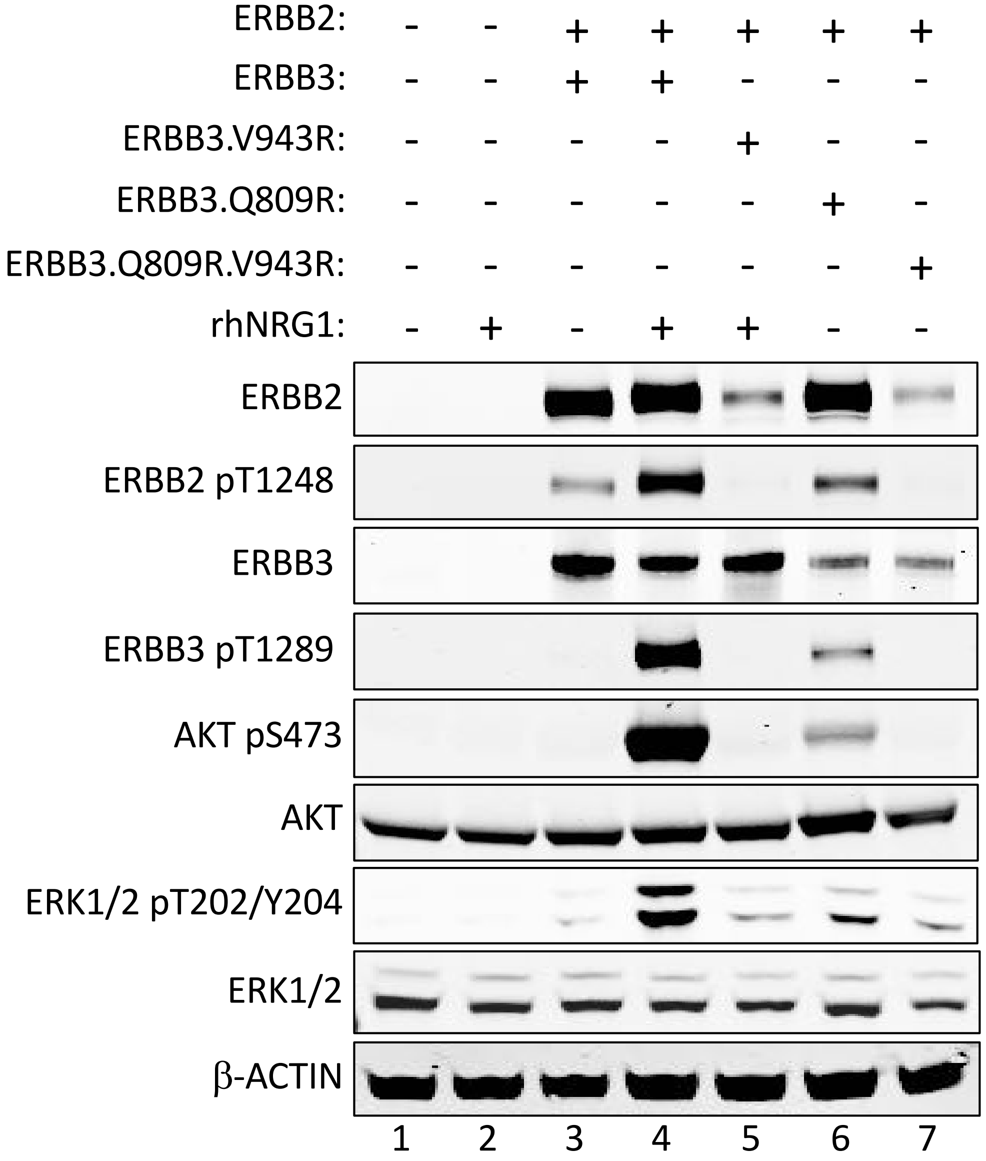

Figure S2. Western blots of canonical ERBB2/ERBB3 receptor signaling readouts. Ba/F3 stable lines were deprived of IL-3 overnight and either left untreated or given rhNRG1 at 100 ng/ml for 15 min prior to lysis.

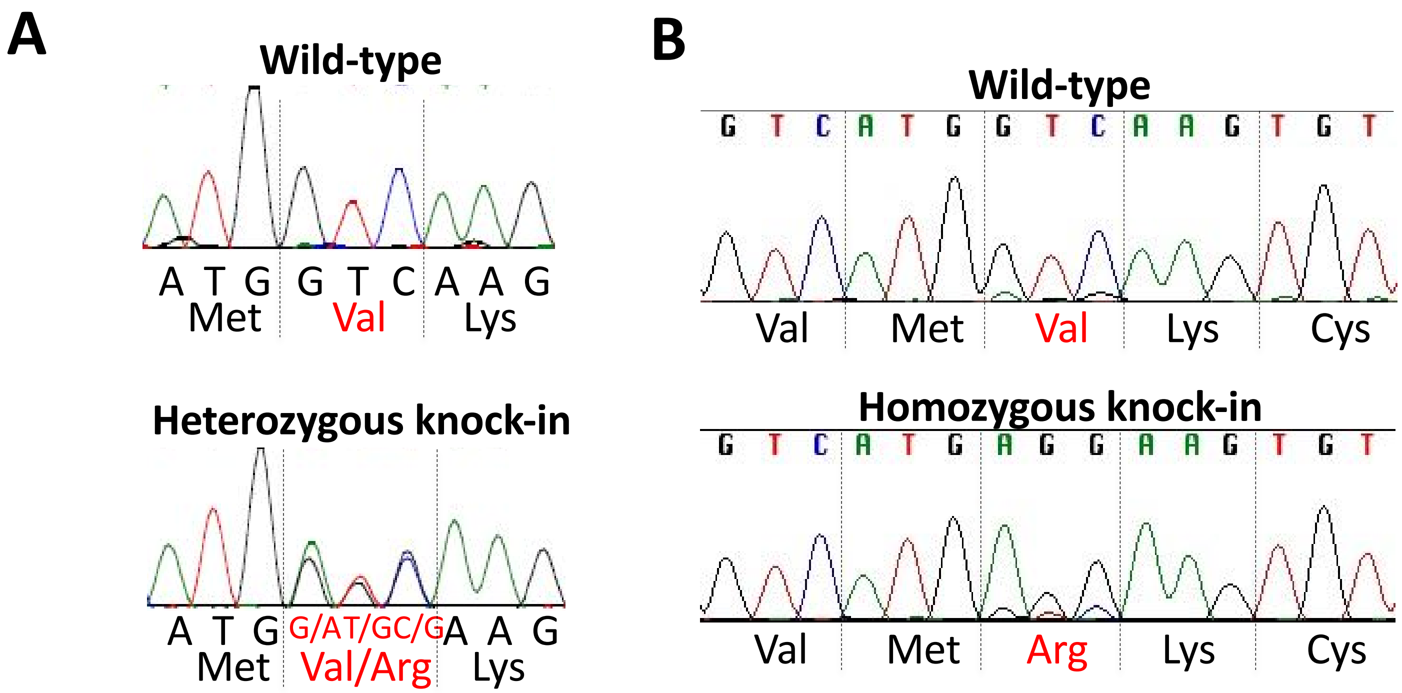

Figure S3. RT-PCR validation of mouse genotypes. A) RNA was extracted for cDNA synthesis from wild-type or heterozygous *Erbb3*$^{V943R/+}$ knock-in embryos. Gene-specific primers flanking the region of interest were used to amplify and sequence the mutation. B) Similar to (A), using epithelial cells isolated from collagenase-digested mammary glands from 6-week old *Erbb3*$^{V943R/V943R;MMTV\text{-}Cre}$ female mice.

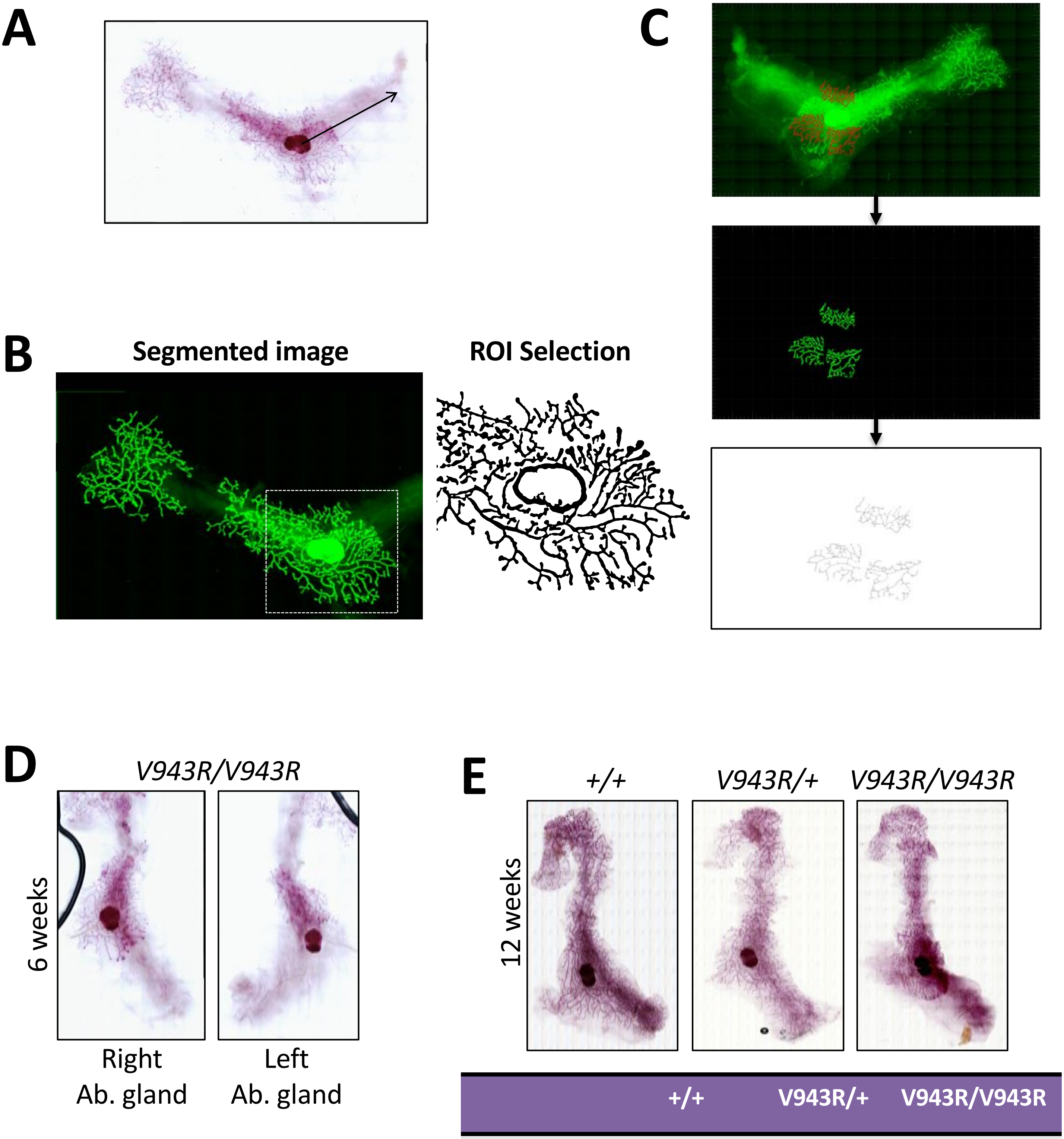

	+/+	V943R/+	V943R/V943R
Full ductal extension	9/9 mice (100%)	5/5 mice (100%)	11/13 mice (85%)
Incomplete extension	0/9 mice (0%)	0/5 mice (0%)	2/13 mice (15%)

Figure S4.

Figure S4. Analysis of mammary gland ductal properties in *Erbb3*$^{V943R/V943R;MMTV\text{-}Cre}$ mice. A) Carmine-stained mammary gland whole mounts were imaged as a collection of tiled images using a 4X objective on a Nikon Ti-E microscope with Fi1 color camera (Nikon Instruments). B) Color images were converted into binary greyscale images by removing color channels and inverting the image to create a monochrome image. The Surfaces function was used to generate the binary mask of the ducts and copied to a new channel. This image was exported to FIJI where a large ROI centered over the lymph node was created and the percent area of the ROI containing the mask was measured. C) To quantify the duct branching 3 identical ROIs were selected from the area surrounding the lymph node of each gland. The Surfaces function was used to generate the binary mask of the ducts contained within each ROI. A gaussian filter was applied to smooth the resulting mask and facilitate skeletonization. The resulting binary image was exported to FIJI and the Skeletonize and Analyze Skeleton 2D/3D functions were used to generate branching statistics. D) Carmine-stained abdominal glands from the same mouse showing different extents of duct elongation. E) Carmine-stained abdominal glands from 12-week old *Erbb3*$^{V943R/V943R;MMTV\text{-}Cre}$ mice. The number and percentage of mice of the indicated genotypes that present an elongation defect is shown. Of the two V943R/V943R mice that showed an elongation defect, a representative image is shown.

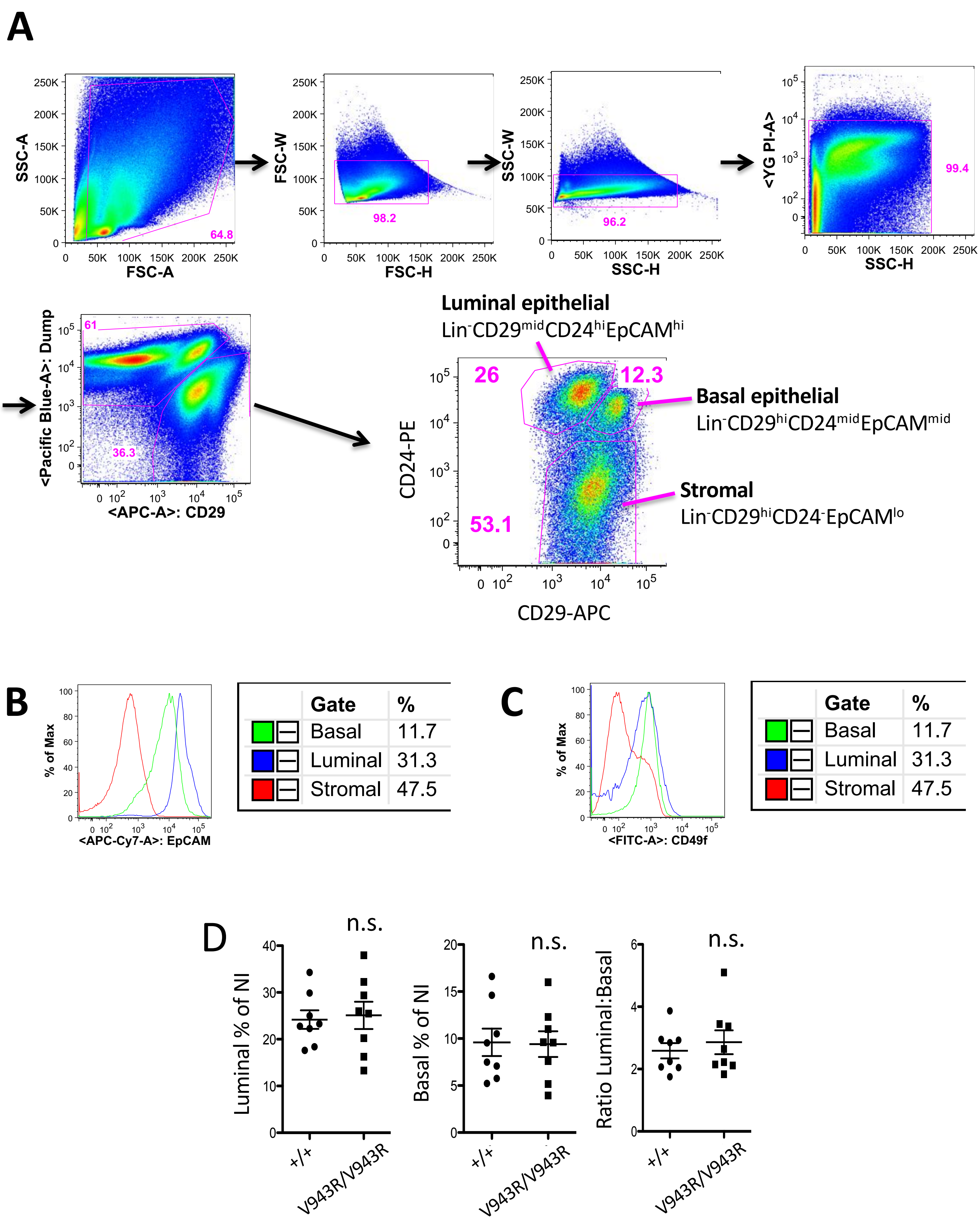
A
SSC-A
FSC-A
64.8
FSC-W
FSC-H
98.2
SSC-W
SSC-H
96.2
<YG PI-A>
SSC-H
99.4
<Pacific Blue-A>: Dump
<APC-A>: CD29
61
36.3
Luminal epithelial
Lin-CD29midCD24hiEpCAMhi
26
12.3
Basal epithelial
Lin-CD29hiCD24midEpCAMmid
Stromal
Lin-CD29hiCD24-EpCAMlo
53.1
CD24-PE
CD29-APC
B
% of Max
<APC-Cy7-A>: EpCAM
Gate %
Basal 11.7
Luminal 31.3
Stromal 47.5
C
% of Max
<FITC-A>: CD49f
Gate %
Basal 11.7
Luminal 31.3
Stromal 47.5
D
n.s.
Luminal % of NI
Basal % of NI
Ratio Luminal:Basal
+/+
V943R/V943R

Figure S5

Figure S5. FACS analysis of mammary gland cell populations in *Erbb3*$^{V943R/V943R;MMTV\text{-}Cre}$ mice. A) Gating strategy for measuring cell populations in 6-week old mammary glands by flow cytometry. B) Relative EpCAM expression by the final gated populations shown in (A). C) Relative CD49f expression by the final gated populations shown in (A). D) Abdominal mammary glands from 6-week old *Erbb3*$^{+/+;MMTV\text{-}Cre}$ (N=8) and *Erbb3*$^{V943R/V943R;MMTV\text{-}Cre}$ (N=8) mice were dissociated with collagenase overnight prior to labeling with an antibody cocktail and analyzed by flow cytometry. Graphs indicate the percentage of Lin-CD29^{+} cells that are luminal epithelial (Lin^{-}CD29^{mid}CD24^{hi}EpCAM^{hi}) or basal epithelial (Lin^{-}CD29^{hi}CD24^{mid}EpCAM^{mid}), as well as the ratio of luminal to basal cells (n.s. = not significant).

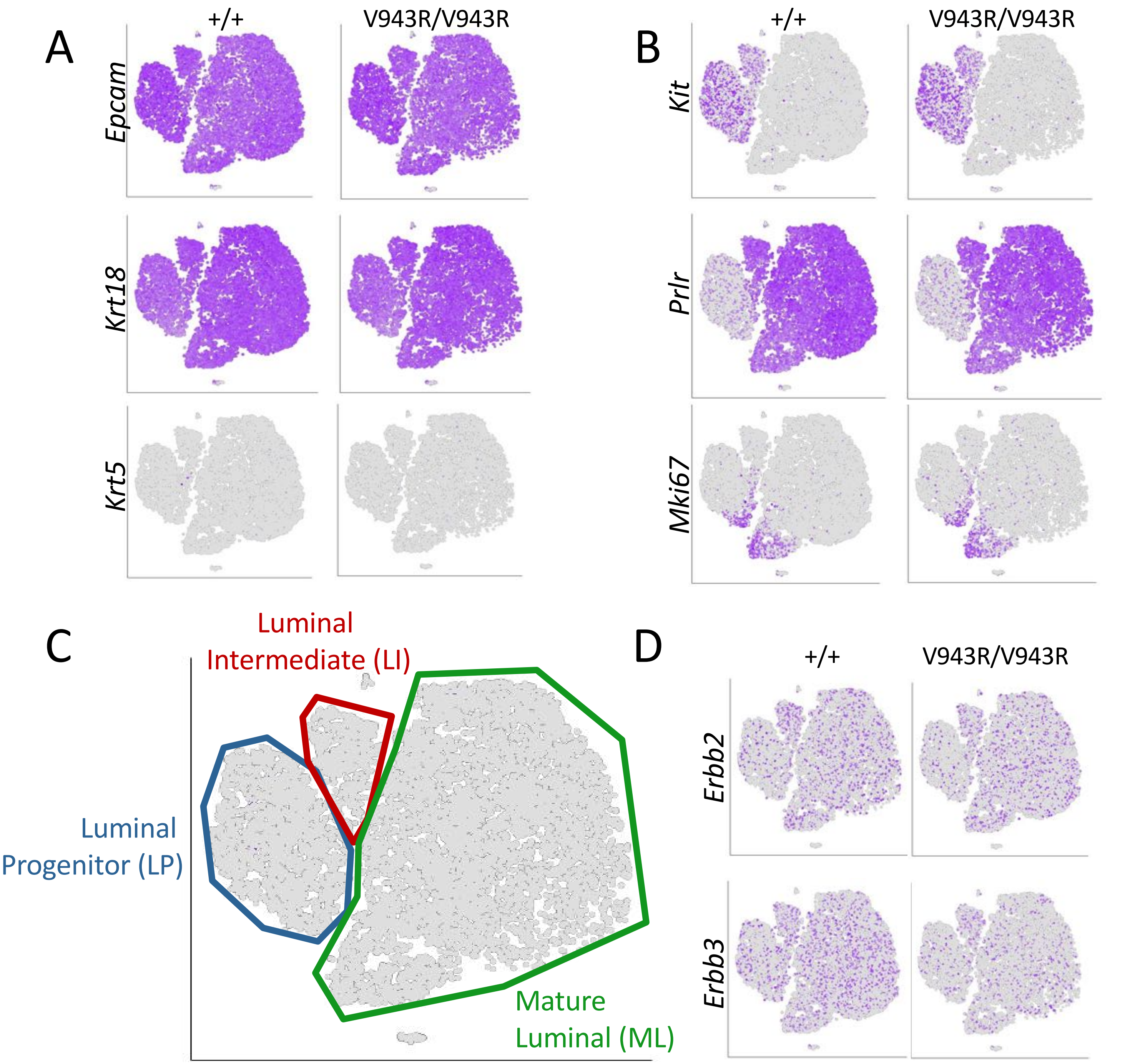

Figure S6. Validation and classification of major luminal epithelial subsets within the single cell dataset. A) Comparison of *Epcam* (pan-epithelial marker), *Krt18* (luminal marker), and *Krt5* (basal marker) mRNA expression. B) Comparison of luminal intermediate markers *Kit* and *Prlr*, as well as proliferation marker *Mki67*. C) Based upon (A) and (B), three major subpopulations are identified. D) Expression of *Erbb2* and *Erbb3*.
