# Supplementary material for: Embryonic lethality and defective mammary gland development of activator‐function impaired conditional knock‐in Erbb3 V943R mice
Source: Adv Genet (Hoboken). 2020 Dec 5;2(1):e10036. doi: 10.1002/ggn2.10036 (PMC9744554; doi:10.1002/ggn2.10036)
Supplement: Supplementary file 2 — Appendix S2: Supporting information [file GGN2-2-e10036-s001.pdf]

ADVANCED GENETICS
Open Access

Embryonic lethality and defective mammary gland development of activator-function impaired conditional knock-in *Erbb3*^{V943R} mice

Kate Senger, Wenlin Yuan, Meredith Sagolla, Jonas Doerr, Brad Bolo, James Ziai, Kai-Hui Sun, Soren Warming, Merone Roose-Girma, Na Zhang, Lucinda Tam, Robert J. Newman, Subhra Chaudhuri, Aju Antony, Leonard D. Goldstein, Steffen Durinck, Bijay S. Jaiswal, Daniel Lafkas, Zora Modrusan, Somasekar Seshagiri*

*Corresponding

Review timeline:	
	Date Submitted: 30-Jul-2020
	Editorial Decision: 03-Sep-2020 Minor Revision
	Revision Received: 15-Oct-2020
	Editorial Decision: 09-Nov-2020 Accept in Principle
	Revision Received: 16-Nov-2020
	Accepted: 16-Nov-2020

Editor: Myles Axton

Initial Editorial Evaluation	30-Jul-2020

Summary

"Expression of Erbb3V943R in the [mouse] mammary gland epithelium using MMTV-Cre [knock-in] results in delayed elongation of the ductal network during puberty." without perturbing the branching of ducts. In contrast Erbb3D850N/D850N;MMTV-Cre knock-in that blocks ATP binding (RTK dead) produces morphologically normal mammary duct structure. The normal developmental role of ERBB3 is allosteric and the Erbb3V943R knock-in reduces the number of a specific fibrinogen-secreting subset of luminal cells.

Scope

Do the research, methods or topics fit within the aims of this, or another journal?

The work is of scope for *Advanced Genetics* and the work can benefit from peer review in its current form, but the study, although complete and well documented, appears to offer a small and highly specialized increment to existing knowledge without specific experiments and discussion about how this model is to be used in understanding mammary development and function, cancer predisposition, oncogenesis and tumor drug therapy resistance.

- The normal developmental role of ERBB3 in mammary ductal structure in mouse has already been established by tissue-specific knock-out. However, interpretation of this knockout is difficult in the context of multiple ERBB3 heterodimers and branching signaling pathways (ERBB2 and EGFR to MAPK and ERBB3 to PI3K). Genetic analysis of ERBB3 ligand independent activation, RTK deficient and allosteric (here V943) mutations seems logical way to address this complexity.
- The work stops with mammary development in the virgin mouse. Lactating, involuting, and tamoxifen treated knock-in mice would be a logical investigation of the importance of ERBB3 signaling and a bridge between developmental and cancer relevance. This work does not directly address the role of ERBB3 allosteric activation or stabilization of ERBB2 in the context of cancer cell identity, proliferation, cancer development in mouse mammary gland or in human, or the acquisition of hormonal therapy, targeted RTK therapy, monoclonal anti-ERBB3 or ERBB2 therapy (Herceptin). Nor does it provide mechanistic interpretation for recurrent ERBB2 or ERBB3 mutations reported in COSMIC. The IL-3 dependent Ba/F3 pro-B cell line used to investigate the signaling proficiency of the mutant ERBB3 alleles is unrelated to either mammary development or breast cancer, or cancer cell therapy resistance. Reporting is limited by use of mouse coordinates and lack of reference to investigation into point mutations in ERBB genes in human breast cancer.

1st Peer Review	03-Aug-2020 to 02-Sep-2020

Reviewer #1 recommends Minor Revision

In this manuscript, Senger et al create a version of ERBB3 with a mutation (V943R) that alters a potentially critical amino acid within the activator interface and examine the effect that this mutant has on cellular signaling and key biologic functions identified previously in conventional Erbb3 knockout mice. The authors first introduced ERBB2 and ERBB3 (wild-type or V943R) simultaneously into IL-3 dependent Ba/F3 cells and asked whether stimulation with the ERBB32 ligand neuregulin-1 (NRG1) could rescue these cells following IL-3 withdrawal—they found that NRG1 rescued the cells in the presence of wild-type ERBB3, but not the V943R mutant. This led the authors to construct mice in which the expression of wild-type Erbb3 could be replaced by Erbb3 V943R in a Cre-inducible fashion. Whole animal expression of Erbb3 V943R beginning at E0.5 recapitulated the previously reported phenotype of Erbb3-/- mice, indicating that V943 is critical for Erbb3 function. Activation of Erbb3 V943R expression with mammary-specific MMTV-Cre showed that the mutant delayed the elongation of mammary ducts. Although it did not alter ductal branching or change the ratio of luminal to basal epithelial cells, the V943R mutant thinned the luminal layer, an affect that single cell RNA-Seq showed was due to reducing the number of a luminal epithelial subpopulation that the authors show is associated with the expression of key transcripts potentially affecting interactions with the

extracellular matrix.

The work presented in this manuscript is well done and supports the claims made by the authors. This work is also likely to be of interest to the readership of Advanced Genetics. While there is little to criticize about the experimental work, there are some points in the text that the authors should address to improve the manuscript:

1) In describing the signaling pathway alterations seen in Ba/F3 cells, the authors refer the reader to Fig. S2 (line 131). It appears that this should point the reader to Fig. S1 instead.
2) In the text, the authors refer the readers to Tables 1 and 2. However, these tables are in the supplemental material, where they are designated as Tables S1 and S2.
3) The star in Fig. 3C is red and difficult to find against the presented histology. It would be helpful to replace this star with a star of a different color that stood out better.
4) Perhaps I'm misunderstanding, but in Supplemental Figure 3E, the figure legend says that "the number and percentage of mice of the indicated genotypes that present an elongation defect is shown". However, the main text seems to say that what is indicated is the number of animals lacking an elongation defect.
5) The sentence spanning lines 348-351 is confusing. It states that "these similarities in phenotype between the knock-out mice, that leads and a complete loss of the protein indicates the activator function of ERBB3…". Please restate this sentence so that its intention is clear.
6) Although the authors have also constructed mice with a Cre-inducible D850N mutation, these animals are not mentioned until the final paragraph of the Discussion and no data is presented to support the authors' statement that this mutant has no effect on mammary duct development. There is also a supplemental figure (Figure 6) showing how the targeting vector was constructed. The authors should provide more data about this mutant or delete it from the paper.

Reviewer #2 recommends Accept

The authors present their findings from experiments aiming to assess the biological effects mediated by ErbB3 V943, a residue of ErbB3 that may be critical to ErbB3 interaction with other ErbB family members. The authors use a knock-in approach for conditional ErbB3-V943R expression under the control of the endogenous Erbb3 allele promoter in mice.The authors find that ErbB3-V943R recapitulates many of the same phenotypes seen upon global ErbB3 knockout (i.e., embryonic lethality) or mammary-specific ErbB3 loss (delayed ductal elongation during puberty), although some differences exist. Further, the authors find that a specific subset of fibrinogen-producing mammary epithelial cells are depleted upon mammary expression of ErbB3-V943R.

Together, these data enrich the understanding of ErbB3 signaling as a potent activator within ErbB heterodimers, while highlighting the potential role that ErbB3 may play as a scaffold.

Reviewer #3 recommends Major Revision

Senger et al. focuses on the V943L ERBB3 mutation, which abrogates ERBB3's ability to activate other RTK's including ERBB2. The authors demonstrate that in ERBB2 expressing Ba/F3 cells, ERBB3 V943R abrogates growth relative to wt ERBB3 or the oncogenic Q809R mutation. The authors generate conditional knock-in mice expressing ERBB3 V943R. They demonstrate that Erbb3V943R/ V943R die prenatally at approximately E12.0 and display defects during cardiac development. The authors further examine ERBB3 V943R in the mammary gland using the mammary epithelial-specific MMTV-Cre driver to express Erbb3V943R allele in the mammary gland. In this context Senger et al. finds that Erbb3V943R/ V943R;MMTV-Cre mice to some extent demonstrate shortened ducts and a reduction in luminal thickness in terminal end buds relative to Erbb3+/+;MMTV-Cre mice. The author then perform single cell RNA-sequencing analysis of luminal epithelial cells from Erbb3+/+;MMTV-Cre or Erbb3V943R/ V943R;MMTV-Cre mice and find a reduction in a subset of fibrinogen-producing luminal epithelial cells. Comments and concerns are listed below.

1. A stronger rationale should be included for exclusively focusing on ERBB3 V943L. This mutation has not yet been found in any cancers. The authors should include a discussion on this and further clinical implications of their work.
2. Are there cerebellar affects in the embryos of conditional knock-in mice expressing ERBB3 V943L as has been seen in ERBB3-/- embryos (Erickson et al., 1997)? Generating ERBB3-/- knock-out mice in parallel with conditional knock-in mice expressing ERBB3 V943L would have been an ideal positive control to compare to conditional knock-in mice expressing ERBB3 V943L. Why do embryos expressing conditional knock-in of ERBB3 V943L show an earlier lethality at E12.0 versus ERBB3-/- embryos (Erickson et al., 1997) with lethality at E13.5?
3. Figures 5 and 6 focus on single cell RNA-sequencing analysis. However, these results are not validated through independent experiments. Are Sox4, Tgm2, Plk2 elevated in mutant cells versus wt ERBB3 cells using RT-PCR or at the protein level?
4. The last paragraph of the discussion mentions generating mice bearing the D850N mutation which is expected to eliminate any kinase activity. The paper would be strengthened by adding these results to Figure 3 to provide further insight into how ERBB3 is essential.
5. The manuscript has disparate data with some figures unrelated to other figures. Figure 1 focuses on Ba/F3 cells, which depend on IL-3 for survival unless expressing oncogenes for example ERBB2 and ERBB3. Why is there variability in expression of ERBB3 (Fig. S1)? This figure focuses on ERBB2 expressing cells, whereas the remainder of the manuscript does not examine ERBB2 expressing cells.

Minor point:
• The text has a few errors indicating figures and tables (for example first paragraph of Results should list Figs. S1 lanes 5 and 7).

1st Editorial Decision	03-Sep-2020

Manuscript entitled "Embryonic lethality and defective mammary gland development in activator function impaired conditional knock-in Erbb3V943R mice" which you submitted to Advanced Genetics, has been reviewed very favorably and minor revisions have been requested. I invite you to respond to the comments appended below and revise your manuscript. I have identified concurrent recommendations from the reviewers and highlighted (in the attached table) those that I believe to make the largest improvement to the revised manuscript, but please do address all of the reviewer comments in your point-by-point reply.

Editor's understanding of the reviews
In light of the reviewers' detailed recommendations to the authors and the overall concerns of all the reviewers with respect to the journal's standards and those of the field, the editor may highlight or prioritize points for the author to address.

Reviewer #1 Minor revision
Recommends acceptance with only textual alterations. The Cre-inducible D850N (kinase dead) mutation is not however properly described.

Reviewer #2 Accept
Recommends acceptance without specific revision requirements

Reviewer #3 Major revision
Recommends major revisions, but these are only asking for completeness in the phenotyping and reporting and can be completed within the scope of a minor revision.

Author's Response to 1st Review	15-Oct-2020

These are the main reviewer recommendations that the editors believe will make the biggest improvement to this article.
Please do address all reviewer comments listed in the decision letter in your point-by-point response
(you may continue this table to do so if you wish). We hope this summary helps you to understand our decision and expedites the revision process.
We value feedback from author and referees alike.

Reviewer comments	Editor recommendation	Author reply	Changes to Manuscript
1.6 Cre-inducible D850N mutation, these animals are not mentioned until the final paragraph of the Discussion and no data is presented to support the authors' statement that this mutant has no effect on mammary duct 3.4 discussion mentions generating mice bearing the D850N mutation which is expected to eliminate any kinase activity. The paper would be strengthened by adding these results to Figure 3 to provide further insight into how ERBB3 is essential.	ED1 Bring D850N into the Results section and figures and explain how this mutation indicates that ERBB3 is essential (for example for proliferation and cell types, but not for ductal morphogenesis).	ERBB3 kinase domain is a bona fide pseudokinase (Biochem. Soc. Trans. (2013) 41, 969–974). While it can still bind ATP its kinase activity if any is several orders of magnitude lower than that of EGFR and ERBB2. The D850N mutant knock-in mice we generated in the conserved DFG loop renders ERBB3 devoid of any residual kinase activity. A recent study reported an ERBB3 knock-in K740M mice (Cancer Science. 2020;111:137–147). This is an alternate kinase dead version of ERBB3. In both the studies the kinase dead enhanced ERBB3 mice are born normally suggesting that the non-kinase scaffolding function of ERBB3 is sufficient for normal development. Though we did not do extensive characterization of the mice born with the D850N, given it had no discernable defects, based on the reviewer recommendations and editorial recommendation we have moved the figure to results section of the paper. We have updated the discussion in the revised MS to cite the recent paper describing the kinase-dead mice.	Moved the results on D850N to the main section as suggested and edited the manuscript.
3.1 A stronger rationale should be included for exclusively focusing on ERBB3 V943L. This mutation has not yet been found in any cancers. The authors should include a discussion on this and further clinical implications of their work.	ED2 This was an editorial recommendation on the original draft and Reviewer 3 reinforces that this would be desirable	The V943R is not a cancer mutation. The ERBB family kinase activation involved a asymmetric dimer where the c-terminal end of the activator kinase subunit interacts with the N-terminal region of the receiver kinase to activate it. The V943 is a critical residue in this interface and mutating it impairs the activator function of ERBB3. We wanted to test the relevance of the activator function of ERBBE3 in vivo. These	We have updated Figure 1A to show the mutants and the activator-receiver interface. Also, edited text to clarify this further.

		mice are an invaluable reagent that can be used to test the in vivo relevance of ERBB3 in activation of ERBB2 in cancer. Such experiments will further clarify the relevance of ERBB3 targeting in ERBB3 driven cancers.	
3.3 Figures 5 and 6 focus on single cell RNA-sequencing analysis. However, these results are not validated through independent experiments.	ED3 If there are replicates or bulk RNA sequencing to support these results, please provide them as supplementary files	Mammary gland single cell experiments were performed by pooling mammary glands from 3 independent animals. So in effect given this and multiple data points from 1000 of cells it provides an element of replication thought not exact.<br>The validation and further characterization of the mutant animal in the context of mammary tumors is appropriate for a followup study	
3.2 Are there cerebellar effects in the embryos of conditional knock- in mice expressing ERBB3 V943L as has been seen in ERBB3-/- embryos (Erickson et al., 1997)<br>….Why do embryos expressing conditional knock-in of ERBB3 V943L show an earlier lethality at E12.0 versus ERBB3-/- embryos (Erickson et al., 1997) with lethality at E13.5?	ED4 These are essential questions that should be answered in the revised text	Embryonic lethality for ERBB3 null mice is between E11.5 and E13.5 in another study (Nature 1997). The exact timing of death can vary by a few days and the timed pregnancy study design can also contribute to the slight variation in the reported timing of embryonic lethality. As for neurological defects we did not attempt to study this as our focus was to develop a mice that expressed ERBB3 activator function deficient mutant that be used to conditionally activate the mutant V923R allele in a tissue specific manner and use it in follow up studies to understand the role of ERBB3 in tumorigenesis. We have added these points in the revised discussion.	We have updated the references cited and updated the text to discuss these points.
3.5 Why is there variability in expression of ERBB3 (Fig. S1)?	ED5 Please explain your choice of cell system and comment on the effect of ERBB2 expression on ERBB3 stability and ERBB3 activity on ERBB2 levels	These are pooled stable Ba/F3 cells and the purpose of these experiments is to confirm that the mutant protein do not signal as expected. We show sufficient surface expression in Fig 1 using FACS. However the western blots in Fig 1S show some variability I particular where productive signaling is not observed – we discuss this in the main text	

Wish to thank the editor and the reviewers for their time and help with improving our work

Reviewer #1

In this manuscript, Senger et al create a version of ERBB3 with a mutation (V943R) that alters a potentially critical amino acid within the activator interface and examine the effect that this mutant has on cellular signaling and key biologic functions identified previously in conventional Erbb3 knockout mice. The authors first introduced ERBB2 and ERBB3 (wild-type or V943R) simultaneously into IL-3 dependent Ba/F3 cells and asked whether stimulation with the ERBB3 ligand neuregulin-1 (NRG1) could rescue these cells following IL-3 withdrawal—they found that NRG1 rescued the cells in the presence of wild-type ERBB3, but not the V943R mutant. This led the authors to construct mice in which the expression of wild-type Erbb3 could be replaced by Erbb3 V943R in a Cre-inducible fashion. Whole animal expression of Erbb3 V943R beginning at E0.5 recapitulated the previously reported phenotype of Erbb3-/- mice, indicating that V943 is critical for Erbb3 function. Activation of Erbb3 V943R expression with mammary-specific MMTV-Cre showed that the mutant delayed the elongation of mammary ducts. Although it did not alter ductal branching or change the ratio of luminal to basal epithelial cells, the V943R mutant thinned the luminal layer, an affect that single cell RNA-Seq showed was due to reducing the number of a luminal epithelial subpopulation that the authors show is associated with the expression of key transcripts potentially affecting interactions with the extracellular matrix.

The work presented in this manuscript is well done and supports the claims made by the authors. This work is also likely to be of interest to the readership of Advanced Genetics. While there is little to criticize about the experimental work, there are some points in the text that the authors should address to improve the manuscript:

1.1 In describing the signaling pathway alterations seen in Ba/F3 cells, the authors refer the reader to Fig. S2 (line 131). It appears that this should point the reader to Fig. S1 instead.

>> we have fixed the error

1.2 In the text, the authors refer the readers to Tables 1 and 2. However, these tables are in the supplemental material, where they are designated as Tables S1 and S2.

>> we have fixed the error

1.3 The star in Fig. 3C is red and difficult to find against the presented histology. It would be helpful to replace this star with a star of a different color that stood out better.

>> we have fixed this

1.4 Perhaps I'm misunderstanding, but in Supplemental Figure 3E, the figure legend says that "the number and percentage of mice of the indicated genotypes that present an elongation defect is shown". However, the main text seems to say that what is indicated is the number of animals lacking an elongation defect.

>> [co-author] please check and fix

1.5 The sentence spanning lines 348-351 is confusing. It states that "these similarities in phenotype between the knock-out mice, that leads and a complete loss of the protein indicates the activator function of ERBB3…". Please restate this sentence so that its intention is clear.

>> we have edited the text to clarify this

1.6 Although the authors have also constructed mice with a Cre-inducible D850N mutation, these animals are not mentioned until the final paragraph of the Discussion and no data is presented to support the authors' statement that this mutant has no effect on mammary duct development. There is also a supplemental figure (Figure 6) showing how the targeting vector was constructed. The authors should provide more data about this mutant or delete it from the paper.

>> we have moved the data to the main part of the paper as suggested

Reviewer #2

The authors present their findings from experiments aiming to assess the biological effects mediated by ErbB3 V943, a residue of ErbB3 that may be critical to ErbB3 interaction with other ErbB family members. The authors use a knock-in approach for conditional ErbB3-V943R expression under the control of the endogenous Erbb3 allele promoter in mice. The authors find that ErbB3-V943R recapitulates many of the same phenotypes seen upon global ErbB3 knockout (i.e., embryonic lethality) or mammary-specific ErbB3 loss (delayed ductal elongation during puberty), although some differences exist. Further, the authors find that a specific subset of fibrinogen-producing mammary epithelial cells are depleted upon mammary expression of ErbB3-V943R.

Together, these data enrich the understanding of ErbB3 signaling as a potent activator within ErbB heterodimers, while highlighting the potential role that ErbB3 may play as a scaffold**.**

Reviewer #3

Senger et al. focuses on the V943L ERBB3 mutation, which abrogates ERBB3's ability to activate other RTK's including ERBB2. The authors demonstrate that in ERBB2 expressing Ba/F3 cells, ERBB3 V943R abrogates growth relative to wt ERBB3 or the oncogenic Q809R mutation. The authors generate conditional knock-in mice expressing ERBB3 V943R. They demonstrate that Erbb3V943R/V943R die prenatally at approximately E12.0 and display defects during cardiac development. The authors further examine ERBB3 V943R in the mammary gland using the mammary epithelial-specific MMTV-Cre driver to express Erbb3V943R allele in the mammary gland. In this context Senger et al. finds that Erbb3V943R/ V943R;MMTV-Cre mice to some extent demonstrate shortened ducts and a reduction in luminal thickness in terminal end buds relative to Erbb3+/+;MMTV-Cre mice. The author then perform single cell RNA-sequencing analysis of luminal epithelial cells from Erbb3+/+;MMTV-Cre or Erbb3V943R/V943R;MMTV-Cre mice and find a reduction in a subset of fibrinogen-producing luminal epithelial cells. Comments and concerns are listed below.

3.1 A stronger rationale should be included for exclusively focusing on ERBB3 V943L. This mutation has not yet been found in any cancers. The authors should include a discussion on this and further clinical implications of their work.

>> we have clarified this – V943L is not a cancer mutation, it rather a mutation the abrogates the activator function of EBBR3

3.2 Are there cerebellar affects in the embryos of conditional knock-in mice expressing ERBB3 V943L as has been seen in ERBB3-/- embryos (Erickson et al., 1997)? Generating ERBB3-/- knock-out mice in parallel with conditional knock-in mice expressing ERBB3 V943L would have been an ideal positive control to compare to conditional knock-in mice expressing ERBB3 V943L. Why do embryos expressing conditional knock-in of ERBB3 V943L show an earlier lethality at E12.0 versus ERBB3-/- embryos (Erickson et al., 1997) with lethality at E13.5?

>> this is discussed in the response above in the table of responses to editor's questions

3.3 Figures 5 and 6 focus on single cell RNA-sequencing analysis. However, these results are not validated through independent experiments. Are Sox4, Tgm2, Plk2 elevated in mutant cells versus wt ERBB3 cells using RT-PCR or at the protein level?

>> this is discussed in the response above in the table of responses to editor's questions. Some of the suggested studies while pertinent is more suitable for a follow up study

3.4 The last paragraph of the discussion mentions generating mice bearing the D850N mutation which is expected to eliminate any kinase activity. The paper would be strengthened by adding these results to Figure 3 to provide further insight into how ERBB3 is essential.

>> we have moved this to the main section as suggested

3.5 The manuscript has disparate data with some figures unrelated to other figures. Figure 1 focuses on Ba/F3 cells, which depend on IL-3 for survival unless expressing oncogenes for example ERBB2 and ERBB3. Why is there variability in expression of ERBB3 (Fig. S1)? This figure focuses on ERBB2 expressing cells, whereas the remainder of the manuscript does not examine ERBB2 expressing cells.

>> ERBB3 requires EBBR2 to signal and needed in BaF3 setting to examine ERBB3 activity – the study is about ERBB3 and hence it's the focus of the manuscript. We have edited the MS to clarify the focus on ERBB3 and the relevance of its activator function

Minor point:

3m1 The text has a few errors indicating figures and tables (for example first paragraph of Results should list Figs. S1 lanes 5 and 7).

>> Thank you pointing this out – we have edited the text and corrected the errors

2nd Peer Review	15-Oct-2020 to 06-Nov-2020

Reviewer #1 recommends Accept

The authors have responded thoroughly and thoughtfully to the previous comments. This significantly improves the manuscript.

Reviewer #3 recommends Accept

The revised manuscript is improved and acceptable for publication.

2nd Editorial Decision	09-Nov-2020

The manuscript has now been seen by two of the original reviewers, and we have now decided to accept the revised manuscript in principle, subject to the attached formatting requirements.

2nd Review and Author's Response	15-Nov-2020

>> Thank the editor and reviewers for the time and effort in reviewing and providing constructive suggestions to help improve the manuscript.
